# Markov State Models: To Optimize or Not to Optimize

**DOI:** 10.1021/acs.jctc.3c01134

**Published:** 2024-01-02

**Authors:** Robert E. Arbon, Yanchen Zhu, Antonia S. J. S. Mey

**Affiliations:** † EaStCHEM School of Chemistry, David Brewster Road, Joseph Black Building, The King’s Buildings, Edinburgh EH9 3FJ, United Kingdom; ‡ Redesign Science, 180 Varick St., New York, New York 10014, United States

## Abstract

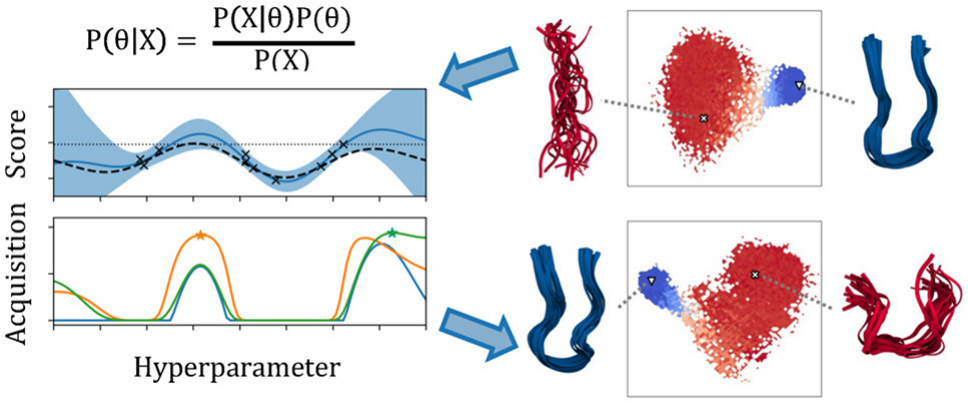

Markov state models (MSM) are a popular statistical method for analyzing the conformational dynamics of proteins including protein folding. With all statistical and machine learning (ML) models, choices must be made about the modeling pipeline that cannot be directly learned from the data. These choices, or hyperparameters, are often evaluated by expert judgment or, in the case of MSMs, by maximizing variational scores such as the VAMP-2 score. Modern ML and statistical pipelines often use automatic hyperparameter selection techniques ranging from the simple, choosing the best score from a random selection of hyperparameters, to the complex, optimization via, e.g., Bayesian optimization. In this work, we ask whether it is possible to automatically select MSM models this way by estimating and analyzing over 16,000,000 observations from over 280,000 estimated MSMs. We find that differences in hyperparameters can change the physical interpretation of the optimization objective, making automatic selection difficult. In addition, we find that enforcing conditions of equilibrium in the VAMP scores can result in inconsistent model selection. However, other parameters that specify the VAMP-2 score (lag time and number of relaxation processes scored) have only a negligible influence on model selection. We suggest that model observables and variational scores should be only a guide to model selection and that a full investigation of the MSM properties should be undertaken when selecting hyperparameters.

## Introduction

Markov state models (MSMs) are a popular model for extracting kinetic information from unbiased molecular dynamics simulations. Recent studies include a wide range of applications, such as understanding protein association kinetics,^1,2^ enzyme dynamics,^3^ ion binding mechanisms,^4,5^ hydrogen bond dynamics,^6^ drug binding mechanisms for drug discovery,^7−11^ mutational effects on conformational dynamics,^12−15^ kinetics of intrinsically disordered proteins,^16^ protein folding,^17^ and understanding allostery.^18−20^ Estimating a MSM proceeds^21^ by first collecting a data set of unbiased molecular dynamics (MD) simulations, then associating each molecular conformation with discrete states, counting transitions between states separated by the temporal resolution of the model (the lag time, τ), and then deriving transition probabilities between states.^22^ The final model is summarized by transition matrix **T**, where the elements *T*_*ij*_ are the conditional probabilities of being in state *i* at time *t* and then transitioning to a state *j* at time *t* + τ: *T*_*ij*_(τ) = *P*(*j*, *t* = *t* + τ|*i*, *t* = *t*). The eigenvectors of the transition matrix represent the dynamic modes of the system as they relax to the equilibrium distribution.

The entire process of transforming MD frames into a transition matrix involves making a number of modeling choices, called *hyperparameters*. Hyperparameters are differentiated from the *parameters* of the model because the latter are calculated from the data via the optimization of a loss function (e.g., the negative log-likelihood), while the hyperparameters are chosen via expert judgment, or via some summary metric of the model.^23^ For MSMs, the important hyperparameters^24−26^ are which subset of atoms from the simulation to include (e.g., a protein loop, pocket, or other substructure of interest), the transformation of these coordinates into important features (e.g., residue–residue distances, backbone dihedral angles), and dimensionality reduction onto a set of important collective variables (typically time-lagged independent component analysis, tICA,^27^ is used for this purpose). Other methods such as PCA,^28^ the generalization of tICA, and relaxation mode analysis^29^ have also been used, but we focus on the most commonly use method (tICA). Finally, we define discrete states from these collective variables (via some clustering algorithm such as k-means). Therefore, the parameters of a MSM are the conditional probabilities in the transition matrix, *T*_*ij*_, whereas the hyperparameters are all of the choices (choice of features, clustering algorithm, etc.) that gave us the specific state definitions used in the likelihood maximization step.

Hyperparameter optimization is an important part of modern statistical and machine learning (ML) analysis pipelines^23,30−32^ as hyperparameters can have a strong impact of the performance of a model. There are several methods to find the optimal set of hyperparameters, from exhaustively searching a uniformly spaced grid of choices^33^ or randomly selected from a predefined search space,^30^ evolutionary and population algorithms,^34−37^ to active learning approaches such as Bayesian optimization.^32,38−40^

No “ground truth” data exist for MSMs used for the analysis of protein MD trajectories, so the accuracy of the eigenvectors cannot be judged absolutely. However, a family of variational scores exists which provides a means to compare the relative accuracy of MSMs and thus allows hyperparameter optimization to be performed. The first score to be developed was the cross-validated generalized matrix Rayleigh quotient,^41^ GRMQ, which pertains to reversible MSMs; while the variational approach to Markov processes (VAMP) scores^25,42^ extended these ideas to both reversible, nonreversible, and nonstationary models. These scores measure how well the eigenvectors of the transition matrix (singular vectors in the case of nonreversible models) approximate the “true” eigenvectors in a variational sense; i.e., the higher the score is, the better the approximation is. Thus, optimization of eigenvectors can proceed without the need for a “ground truth” to compare to.

To use a variational score, it is necessary to specify the lag time (τ) of the MSM and the number of slow relaxation modes to optimize (*k*), and then estimate the MSM with different hyperparameters. The “best” set of hyperparameters is the one with the highest variational score. In the case of the VAMP-E score, one may also add *k* to the list of hyperparameters to optimize.

This procedure removes the need for potentially arbitrary hyperparameter selection, with the concomitant risk of findings that are not robust to changes in modeling assumptions. This method has been used in several different studies.^24,43−51^ In addition, it has allowed investigations into the roles of various hyperparameters and other methods for hyperparameter selection to be developed. In Husic et al.,^51^ the authors used the GMRQ to show that the Ward and k-means methods are optimal for clustering conformations for MSMs. In Husic et al.,^24^ the authors performed a sensitivity analysis of the GMRQ in order to determine the sensitivity of hyperparameters in describing protein folding. An extension of the VAMP score by Scherer et al.^25^ showed that the optimal set of features could be selected before going through the full MSM creation and scoring pipeline. However, using variational optimization is not the only method for the principled construction of MSMs, see, for example, Nagel et al.’s^28,52^ careful construction of a MSM of the Villin headpiece. In this work, the authors stress the need for models to produce explanations with sufficient spatial and temporal resolution to resolve metastable states and folding mechanisms. In addition, they note the need to calibrate against different physical conditions, such as temperature.

It is tempting to think that with a single model metric and state-of-the-art ML optimization software, it should be possible to form an automatic pipeline wherein simulation data are fed in and a single optimized MSM describing the kinetics and thermodynamics of the system emerges. However, many detailed questions must be answered before such a pipeline is possible. First, do variational scores refer to the same relaxation mode across all possible combinations of hyperparameters? It is possible that with certain combinations of hyperparameters, the eigenvectors could describe different relaxation modes. It is therefore possible that the variational scores do not compare the same set of processes across different sets of hyperparameters. Second, the MSM lag time and number of scored processes interest will affect the variational scores—does this have any material effect on how we rank different sets of hyperparameters? Third, do we need variational scores to optimize models at all? Will model observables, such as the implied time scales, suffice to optimize MSMs? Finally, does hyperparameter optimization work for MSMs compared with randomly sampling hyperparameters? Here, we use a common method (Bayesian optimization with tree Parzen estimators) for optimizing machine learning models to find the optimal hyperparameters.

The remainder of this work is structured as follows. In the next section, we cover the necessary theory to understand MSMs and Bayesian optimization of hyperparameters; this is followed by the methods and results analysis. The review concludes with some recommendations based on our findings.

## Theory

### Markov State Models

#### Overview

What follows is a brief overview of the theory of Markov state models (MSMs), for a more detailed picture, see some of the many good references.^22,26,53^ MSMs describe the first-order conformational kinetics of a system by specifying the conditional probability of transitioning from a state *i* at a time *t* to a state *j* at a time *t* + τ later. This information is summarized in the transition matrix *T*_*ij*_(τ) = *P*(*j*, *t* + τ|*i*, *t*). Each state, *i*, is a collection of conformations that have similar kinetic properties. The transition matrix is a finite and discrete representation of the underlying Markovian transfer operator, , which describes the dynamics of the system. The first left eigenvector, ϕ_1_, (in descending order of eigenvalue λ_*i*_, with λ_1_ = 1) corresponds to the stationary or equilibrium distribution, which we also label π. The second left eigenvector, ϕ_2_, corresponds to the slowest conformational relaxation process; the third is the next slowest relaxation process and so on. The corresponding right eigenvectors ψ_*i*_ are normalized by π (so ψ_1_ = 1 for all states). The eigenvalues are related to the time scales of these relaxation processes by *t*_*i*_ = −τ/log λ_*i*_. The transition matrix is said to be reversible if it obeys the detailed balance π_*i*_*T*_*ij*_ = π_*j*_*T*_*ji*_.

The transition matrix is specified with respect to a set of *p* basis states, χ_1_, χ_2_, ..., χ_*p*_ which we denote as a vector **χ**. In what follows, the basis states are assumed to be discrete and orthonormal, and each one corresponds to a small region of conformational space. Each frame of an MD trajectory can be mapped to one of these basis states, and these discretized MD trajectories form the data from which the transition matrix is estimated.

The mapping between the atomic coordinates **x** and the basis states we call *f*(**x**; **θ**) = **χ** where **θ** is a vector of parameters of that mapping. For example, *f* may involve projecting coordinates onto the dihedral angles of the backbone of a protein, followed by clustering into 100 discrete states using k-means clustering. The MSM is then specified with a lag time of 10 ns. The parameters of the MSM are the 100 × 100 = 10,000 elements of **T**, while the hyperparameters are **θ** = (backbone dihedrals, k-means, 100) where the elements correspond to the feature, clustering method, and number of basis states, respectively.

#### Estimating a Reversible MSM

The first step in estimating a reversible MSM is projecting the MD trajectories onto the proposed basis states, **χ**. Transitions between each basis state at time *t* and time *t* + τ are tabulated in a count matrix, **C**_0*t*_ (the subscript 0 and *t* refer to the fact that the counted transitions are between *t* and *t* + τ). The population of each state is given by the diagonal matrix, **C**_00_ calculated as the row-sum of the count matrix . A *nonreversible* transition matrix is then given by **T**^irrev^ = **C**_0*t*_**C**_00_^–1^. It is nonreversible because the finite amount of simulation data will not be in perfect equilibrium. A transition matrix and stationary vector which obey a detailed balance, **T**^rev^ and **π**^rev^, can be estimated from **C**_0*t*_ using maximum likelihood estimation with constraints.^22^ The constraints ensure that a detailed balance is obeyed by **T** and its dynamics are reversible. However, once **T**^rev^ and **π**^rev^ have been estimated, they are now inconsistent with **C**_0*t*_ and **C**_00_, as obtained from the MD trajectory.

#### Variational Scores

The key idea behind variational scores is that approximations to the true eigenvectors of the transition matrix will give rise to eigenvalues which are bounded from above by the true eigenvalues, specifically:^41,42^
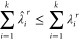
1where λ̂ are the eigenvalues estimated from an approximate basis set **χ** and λ are the true eigenvalues. The sum runs over the first *k* eigenvalues, which are typically the dominant slow relaxation processes that one is interested in approximating, while *r* is some arbitrary positive integer.^42^

When *r* = 1 and the model is assumed to be stationary,^41^ the left-hand side of eq 1 is known as the Generalized Matrix Rayleigh Quotient (GMRQ):
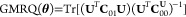
2where **U** is the matrix of eigenvectors of **T**. The functional dependence of the GMRQ on **θ** emphasizes that the eigenvectors and count matrices are dependent on the hyperparameters.

The variational approach to Markov processes placed reversible and stationary MSMs in a broader context of Koopman models, which may or may not be reversible or stationary. In this context, there is a family of variational scores, differentiated by a positive integer *r*:
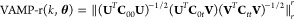
3where **C**_*tt*_ is the column sum of the count matrix ; **U** and **V** are the left and right singular vectors of the transition matrix. The functional dependence on **θ** comes from its influence on the basis states, which in turn determine the singular vectors; *k* is the number of singular vectors being scored and determines the dimensions of **U** and **V**.

The matrix norm denotes the *r*th power of the Schatten-r norm: where
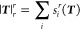
4and *s*_*i*_ are the singular values of a matrix, ***T***.

If the data are stationary, reversible, and *r* = 1, this is equivalent to the GMRQ. With *r* = 2, this expression measures the kinetic variance^54^ captured by the basis sets. The VAMP scores have also been adapted to score models based solely on the type of feature alone (rather than scoring the entire MSM).^25^

As time scales are monotonic functions of the eigenvalues, maximizing the sum of the time scales also maximizes the VAMP scores.

#### Cross-Validation and Bootstrapping

Hyperparameters should be chosen to maximize the performance of a model on unseen data. Simply maximizing the variational score on the data used to fit the model (training data) may result in eigenvectors that describe these data well but do not generalize to new data generated by the same system. This is known as overfitting and is a well-documented phenomenon.^55^ To overcome this problem, the estimated VAMP scores should be close to those attained on unseen data. One estimation method is to withhold a portion of the data (the test set) and calculate the variational scores on this set. While accurate, it requires ignoring a large proportion of the data for training purposes, which may be wasteful when there are only a handful of observed transitions that we are interested in modeling.

Two other popular methods, which make more efficient use of the available data, are cross-validation^56^ and bootstrapping.^57^ The estimators for the variational scores (eqs 2 and 3) were both adapted to be used with cross-validation:^41,42^ data are randomly split into two equally sized subsets. The eigenvectors **U**/**V** are calculated on one set, while the count matrices **C**_00/0*t*/*tt*_ are calculated on the other set. This is repeated *N*_*c*_ times (e.g.,^25^*N*_*c*_ = 50), and an average of the VAMP scores is taken.

The bootstrap does not require a reformulation of estimators. Instead, a number, *N*_*b*_, of new data sets are created from the original data set (e.g.,^57^*N*_*b*_ = 100–1000) and the mean or median of variational scores on each of these data sets used. To create the bootstrapped data sets, trajectories are split into small independent subtrajectories. The subtrajectories are sampled *with replacement* to create a new bootstrapped data set of the same size as the original.

### Hyperparameter Optimization

#### Methods for Optimizing Hyperparameters

Finding the best set of hyperparameters **θ** using either the VAMP scores or implied time scales (we use the term *response* generally) is a black box optimization problem. It is a black box because we do not have access to the gradients ∇_**θ**_VAMP-r(*k*, **θ**), which would facilitate gradient-based optimization. There are three broad classes of optimization techniques in this case: exhaustive searching, model-based searching, and population-based algorithms.

Examples of exhaustive searching are grid search, where hyperparameters are taken from a uniformly placed grid over the hyperparameter search space, and random search, where hyperparameters are randomly sampled from the search space.

Grid search is an effective strategy when the response is sensitive to all of the hyperparameters. However, it has poor scaling with the number of hyperparameters (*N*^*d*^, where *N* is the number of grid points per hyperparameter, and *d* is the number of hyperparameters), so when only a small subset of hyperparameters are relevant, random search is more efficient.^30^

Model-based search algorithms construct surrogate models of the mapping between the hyperparameters and the model response, which are cheap to evaluate and optimize, and use these models to guide hyperparameters to test. Examples include Bayesian optimization with either a Gaussian process or a tree Parzen estimator (TPE) as the surrogate model.^32^ The third class of optimization algorithms is population algorithms, which include evolutionary algorithms,^34,36^ particle swarm optimization,^35,36^ and covariance matrix adaption;^37^ these will not be explored here further.

#### Bayesian Optimization with Tree-Structured Parzen Estimators

We chose tree-structured Parzen estimators to perform optimization because they easily handle numerical as well as categorical hyperparameters and can easily model conditional hyperparameter search spaces (i.e., choosing hyperparameters based on the choices of other hyperparameters); this latter feature is the “tree structure” referred to in the name of the method.

Bayesian Optimization with TPE proceeds as follows.

1. Randomly sample a small set of hyperparameters and measure the response of the resulting MSMs. This gives a hyperparameter trial data set  where *y* is the model response.
2. Construct a model of the probability of the hyperparameters, given the response *p*(**θ**|*y*) as two separate probability density functions:
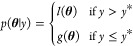
5where y* is some user-specified quantile, γ, of the observations. *l* and *g* are probability models of the “good” and “bad” hyperparameters, respectively, and are explained more fully below.
3. To find the *n* + 1th value of **θ**, we maximize the *Expected Improvement*: .
4. Evaluate **θ**_*n*+1_ on the MSM and measure the response, *y*_*n*+1_, and add (θ_*n*+1_,*y*_*n*+1_) to the hyperparameter trial data set.
5. Repeat steps 2 to 4 until convergence in the maximum value of *y* is reached.

The functions *l* and *g* are Parzen estimators, otherwise known as kernel density estimators. These model the probability density of **θ** by placing truncated Gaussian distributions over each observation of a continuous hyperparameter and a categorical distribution proportional to the observed counts of each level for each discrete hyperparameter. More details can be found in Bergstra et al.^32,40^

This method can also be extended to dual objective functions, that is, when two (or more) responses are optimized, **y**_*i*_ = (*y*_*i*_^1^,*y*_*i*_^2^), for the same model. In this case, the “best” solutions form a Pareto set. Any member of a Pareto set, **y**_*k*_, has both responses superior to all other trials (*y*_*k*_^1^ > *y*_*i*_^1^, *y*_*k*_^2^ > *y*_*i*_^2^ for all *i*) but which are only superior to other members of the set in one response ( or  for *k* and *k*′ in the Pareto set). For dual-objective optimization, the acquisition function was the expected hyper-volume improvement.^58^ This function tries to find hyperparameters that expand the Pareto set. The splitting of observations into two sets is complex, see Ozaki et al.^58^ for details on the splitting algorithm.

## Methods

To answer our research questions, we estimated a large number of Markov state models with different hyperparameters, measured their observables, and analyzed the results. The workflow is summarized as follows. We used existing molecular dynamics trajectories of Chignolin and BBA and fit MSMs with 140 randomly sampled hyperparameters (*hyperparameter trials*) and recorded implied time scales, eigenvalues, and VAMP-2 scores for a range of different lag times (τ). Each observable was estimated with confidence intervals by using bootstrapping. This data constituted our *hyperparameter trial data set* and was analyzed in the first, second, and last results subsections. A “toy” three-state MSM model was constructed to highlight issues with the VAMP-2 score, for reversible transition matrix estimation. We then performed Bayesian optimization with a TPE surrogate function with a variety of different objective functions and used the hyperparameter trial data set to initialize the surrogate function. These results are discussed in the third part of the results.

### Molecular Dynamics

We use simulation data of the fast-folding proteins Chignolin (CLN025) and BBA, two of the 12 fast-folding proteins that have become the *de facto* benchmark data set for testing molecular kinetics methods. The methods used to create this data are described elsewhere.^59^ Important information on the data is shown in Table 1: the average folding time was calculated by the authors;^59^ the subtrajectory length and number of subtrajectories correspond to the data splitting used in the bootstrapping procedure.

**Table 1:** Description of Molecular Dynamics Data

Name	PDB	Simulation time (μs)	Average folding time (μs)	No. residues	Subtrajectory length (μs)	No. subtrajectories
BBA	1FME	325	18	28	2	164
Chignolin	5AWL	106	0.6	10	2	53

### Markov State Models

MSMs were estimated using PyEMMA version 2.5.7^60^ and used a standard pipeline when focusing on the slow relaxation processes:^21,26^

1. Project molecular dynamics (MD) trajectories onto a set of features.
2. Reduce the dimension of the feature trajectories using tICA with a lag time τ_tICA_ by projecting onto the first *m* tICA coordinates.
3. The frames of the tICA trajectories were clustered using the k-means algorithm into *n* discrete microstates.
4. A reversible, maximum likelihood MSM was then estimated.

To save on memory and compute resources, the data were subset in parts of the MSM estimation. The MD trajectories were first strided so that the time between each frame was 1 ns in line with previous analysis in the literature.^61,62^ The cluster centers were estimated on frames separated by 10 ns, i.e., only the zeroth, 10th, etc. frames were used for estimating the cluster centers.

The uncertainty for model observables was estimated using a bootstrap with 100 bootstrap samples. The point estimate and error bars were calculated as the median, 2.5%, and 97.5% quantiles of the distribution from the bootstrap samples.

### Hyperparameters and Scoring

Here, 140 different hyperparameters were randomly sampled from the search space described in Table 2. Each set of hyperparameters and their corresponding model observables are known as a *hyperparameter trial*. Three different features, *f*, were used:

1. dihedrals feature (“dihed.”): the sine and cosine of the ϕ, ψ, and χ_1–5_ angles of the amino acid residues.
2. contact distance feature (“dist.:): the distance between all pairs of residues separated by three or more residues.
3. logistic distance feature (“logit(dist.)”): the same as feature 2 but with a logistic transform applied to the distance (*d*): logit(*d*) = [1 + exp(*s*(*d* – *c*))]^−1^, where center, *c*, and steepness, *s*, have units of Å and Å^–1^, respectively.

**Table 2:** Hyperparameter Search Space^a^

**Features** (*f*)				
Dihedral angles	WHICH			
	*dihed*. = ϕ, ψ, χ_1_, ..., χ_5_ (sine and cosine transformation)			
Contact distances	DEFINITION (*d*)	TRANSFORM	CENTER (*c*, Å)	STEEPNESS (*s*, Å ^–1^)
	•*X*-*X*	•logit(*dist*.)	3 to 15	0.01 to 5
	•Cα-Cα	•*dist*.		
**Decomposition**	EIGENVECTORS (*m*)	LAG TIME (τ_*T*_, ns)	SCALING	
tICA	1 to 20	1 to 100	λ	
				
**Clustering**	CLUSTERS (*n*)			
k-means	10 to 1000			

a *X*-*X* and Cα-Cα refer to the closest heavy atom and α-carbon scheme respectively, for measuring the contact distance (*dist*.). The sine and cosine of dihedral angles were used as features.

The logistic distance feature may be described as a “soft” or “fuzzy” contact map: it takes on the value 0 for *d* ≫ *c* and a value of 1 for *d* ≪ *c* and varies between these two extremes in the neighborhood of *c* with a steepness determined by *s*. The definitions of the contact distances (*d*) were either the closest heavy-atom distance (*X*-*X*) or the distance between the α-Carbons (Cα-Cα). The tICA eigenvectors were scaled by their eigenvalues (λ) so that distances in tICA space correspond to kinetic distances.^54^

The number of trials was approximately proportional to the number of hyperparameters for each feature: 20 trials for the dihedral feature, 40 for the contact distances (20 for each value of the contact distance scheme: *X*-*X*, Cα-Cα), and 80 for the logistic transformation of contact distances (which, in addition to the two distance scheme values, has two other hyperparameters, *c* and *s*).

For each trial, **θ** = (*f*, τ_T_, *m*, *n*, *c*, *s*), a MSM was estimated using the procedure above with a range of Markov lag times, τ: 1, 11,···, 101 ns. For each combination of **θ** and τ, the slowest 2 to 21 eigenvectors were scored using the VAMP-2(*k*, **θ**) (eq 3) and VAMP-2_eq_(*k*, **θ**) score (eq 6):
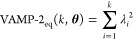
6where λ are the eigenvalues of the MSM transition matrix which obey detailed balance, along with the implied time scales, *t*_*i*_. Each of these observations was estimated as the median of *N*_*b*_ = 100 bootstrapped samples.

VAMP-2(*k*, **θ**) and VAMP-2_eq_(*k*, **θ**) are abbreviated as VAMP2(*k*) and VAMP2_eq_(*k*) from here on, with the dependence on **θ** being assumed.

Selected models were validated by:

1. inspection of structures sampled from microstates which had the most extreme values of ψ_2_.
2. inspection of both ψ_2_ and a two-state coarse-grained model in the space of the first two tICA components.
3. a plot of the mean first passage time as a function of the lag time (as suggested in Suárez et al.^63^).
4. implied time scales as a function of the lag time, τ.

The validation details of the selected models can be found in the Supporting Information.

The hyperparameter trial data set, , consisted of 100 bootstrap samples of 140 unique sets of hyperparameters, at 10 different lag times, with 20 measurements of the implied time scales and 20 measurements of the VAMP2(*k*) score and 20 measurements of the VAMP2_eq_(*k*) score. The total number of these observations (*t*_*i*_, VAMP2(*k*), VAMP2_eq_(*k*)) is therefore 8,400,000.

### Markov Lag Time

The Markov lag time, τ, was calculated from the total hyperparameter trial data set. For each trial, the following gradient was calculated:
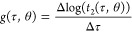
7

The selected Markov lag time, τ*, was chosen as
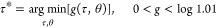
8

**Figure 1:**
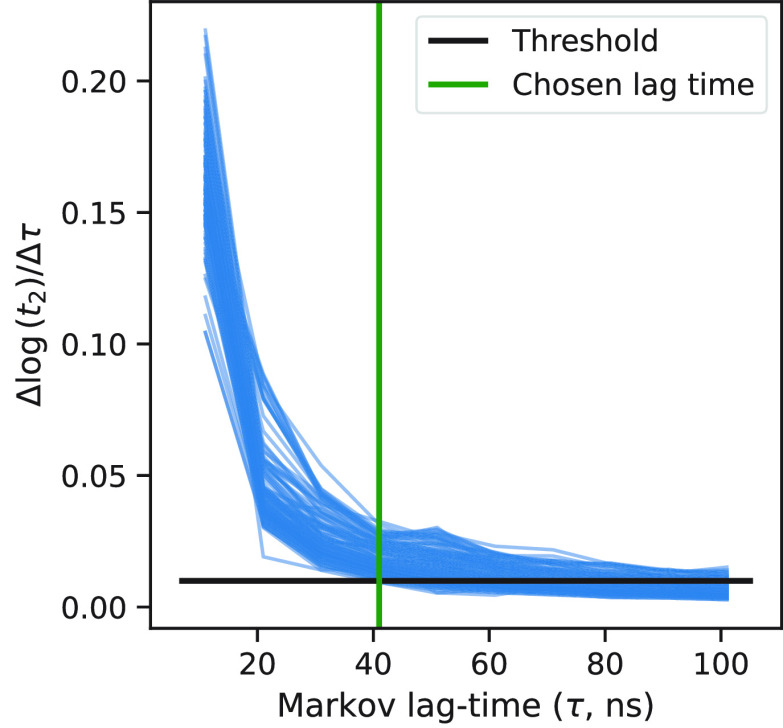
Markov lag time selection. Each blue line is the median of the gradient defined in eq 7 taken over the bootstrapped samples. The black horizontal line is the convergence threshold. The green vertical line is the selected lag time. The data represented here are from BBA; the same method applies to Chignolin.

A graphical representation of this process is shown in Figure 1.

This codifies and extends the generally accepted process by which the implied time scales *t*_*i*_ as a function of τ are plotted on a log scale, and the smallest τ for which *t*_2_ is constant is chosen. Our extension is that we consider a range of different values of θ.

### Optimization

We used Bayesian optimization to optimize the full hyperparameter feature space described in Table 2. We used the tree-structured Parzen estimator as the surrogate function, as implemented in the Python package *Optuna* version 3.0.3^64^ which was also used to perform the optimizations. The optimization runs were initialized with data from the randomly sampled hyperparameter trial data set. The objective functions were estimated as the medians from 20 bootstrap samples. Four different objective functions were used for each protein, two single objective and two dual objective functions, these were

1. *t*_2_: the time scale dominant process.
2. VAMP2_eq_(2) = 1 + λ_2_^2^: the “equilibrium” VAMP2 score of the second (dominant) process.
3. *t*_2_ and *t*_2_/*t*_3_: a multiobjective function of the time scale of the dominant process and the gap between the second and third time scale.
4. VAMP2_eq_(2) and VAMP2_eq_(2)/VAMP_eq_(3): a multiobjective function of the equilibrium VAMP2_eq_(*k*) score of the second process and the gap between the second and third process.

In the case of single objective optimization, the acquisition function was the *expected improvement*; in the case of multiobjective optimization, the acquisition function was the *expected hyper-volume improvement*.^58^ The quantile for splitting observations into “good” and “bad” trials was set at 25%. This information is summarized in Table 3. The number of initial observations is less than the full 140 hyperparameter trials because (a) some trials failed to converge a MSM, and (b) in the case of Chignolin, some MSMs did not have a resolvable value of *t*_3_.

**Table 3:** Hyperparameter Optimization Tasks

Protein	Objective functions	Initial data	No. trials
Chignolin	*t*_2_	131	95
Chignolin	*t*_2_, *t*_2_/*t*_3_	55	141
Chignolin	VAMP2_eq_(2)	131	100
Chignolin	VAMP2_eq_(2), VAMP2_eq_(2)/VAMP2_eq_(3)	55	150
BBA	*t*_2_	136	100
BBA	*t*_2_, *t*_2_/*t*_3_	136	100
BBA	VAMP2_eq_(2)	136	100
BBA	VAMP2_eq_(2), VAMP2_eq_(2)/VAMP2_eq_(3)	136	100

The code used to create the hyperparameter trial data set,  can be found at https://github.com/RobertArbon/msm_sensitivity, and the code used to perform all other analyses can be found at https://github.com/RobertArbon/msm_sensitivity_analysis.

## Results and Discussion

Having created the hyperparameter trial data set, we first highlight some inconsistencies in the VAMP2 scores; then, we show results for optimization using random selection and Bayesian optimization. Finally, we determine what effect the lag time and number of scored eigenvectors have on model selection.

### VAMP2(*k*) Scores of Reversible MSMs Give Inconsistent Results

The VAMP2(*k*) score^42^ provides a principled metric for optimizing MSM hyperparameters. The benefits are that it can be used for stationary, nonstationary, reversible, and nonreversible MSMs. It is directly related to the kinetic variance captured by the basis states such that maximizing the VAMP2(*k*) score will maximize the time scales of pertaining to the first *k* eigenvectors of the model. In addition, it can be used with bootstrapping and cross-validation techniques for assessing generalizability.

**Figure 2:**
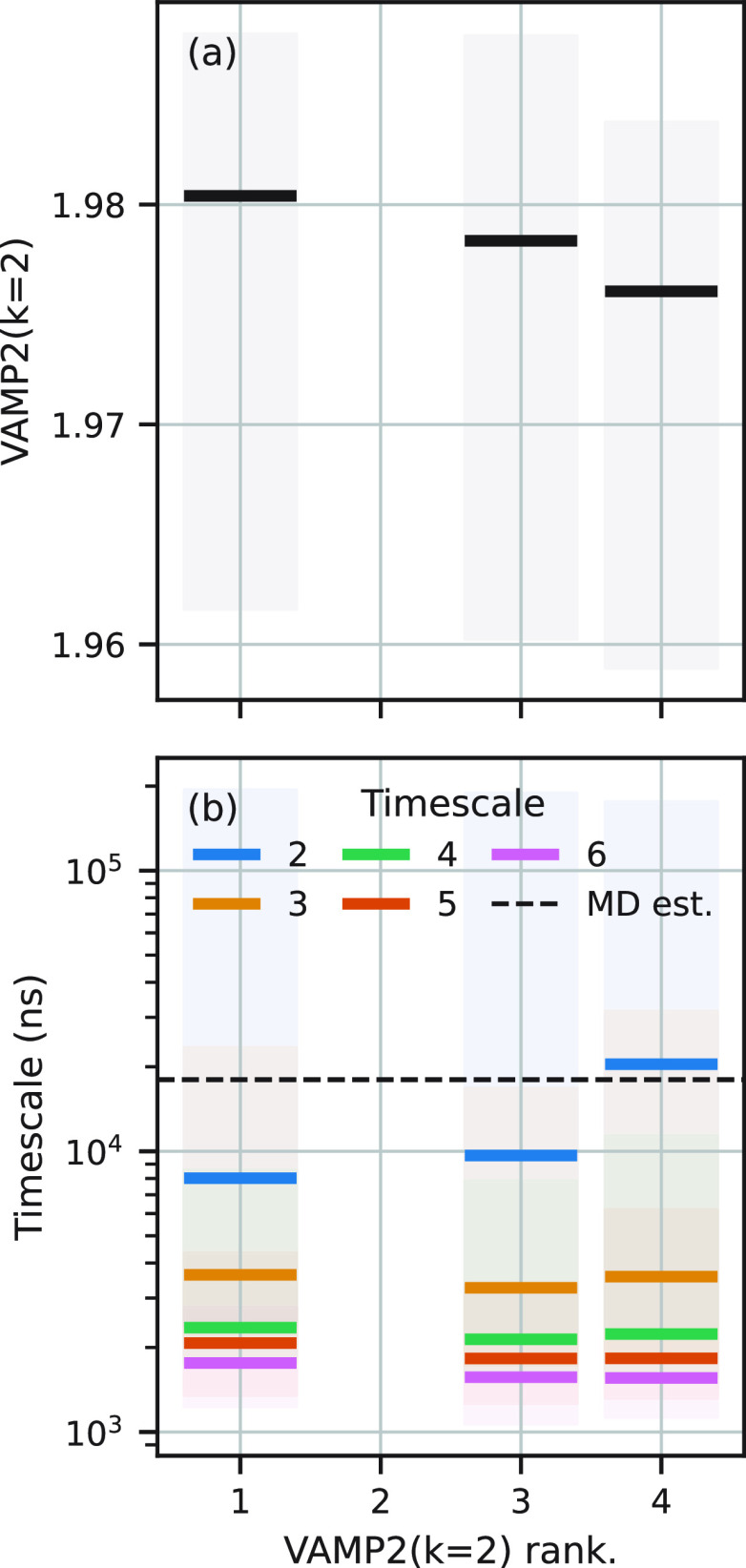
Models with VAMP2(*k*) scores inversely proportional to time scales. (a) VAMP2(2) scores and (b) first five dominant time scales, for a selection of models of BBA. The horizontal axis in both panels is the model rank as judged by the VAMP2(2) score. The selection shows models where the slowest time scale is inversely proportional to the VAMP2(2) score. Models which do not show this correlation are not shown.

Inspection of the VAMP2(*k*) and *t*_2_ values in the hyperparameter trial data set for BBA revealed that for some subsets of the trials, VAMP2(2) was inversely proportional to *t*_2_. An example of this is shown in Figure 2. In panel (a), the VAMP2(2) score is shown for the trials ranked first, third, and fourth. In panel (c), the first five time scales are shown for each model. Time scales for the third to sixth eigenvectors are similar for each trial; however, *t*_2_ clearly *increases* with *decreasing* VAMP2(2) score. The second-ranked model has been omitted for clarity because it does not follow this pattern. We suggest the reason for this behavior is due to the fact by enforcing reversibility in the estimation of the transition matrix it is difficult to get numerical consistency between the three count matrices (**C**_00/0*t*/*tt*_) and the eigenvectors (**U**/**V**) in eq 3.

**Figure 3:**
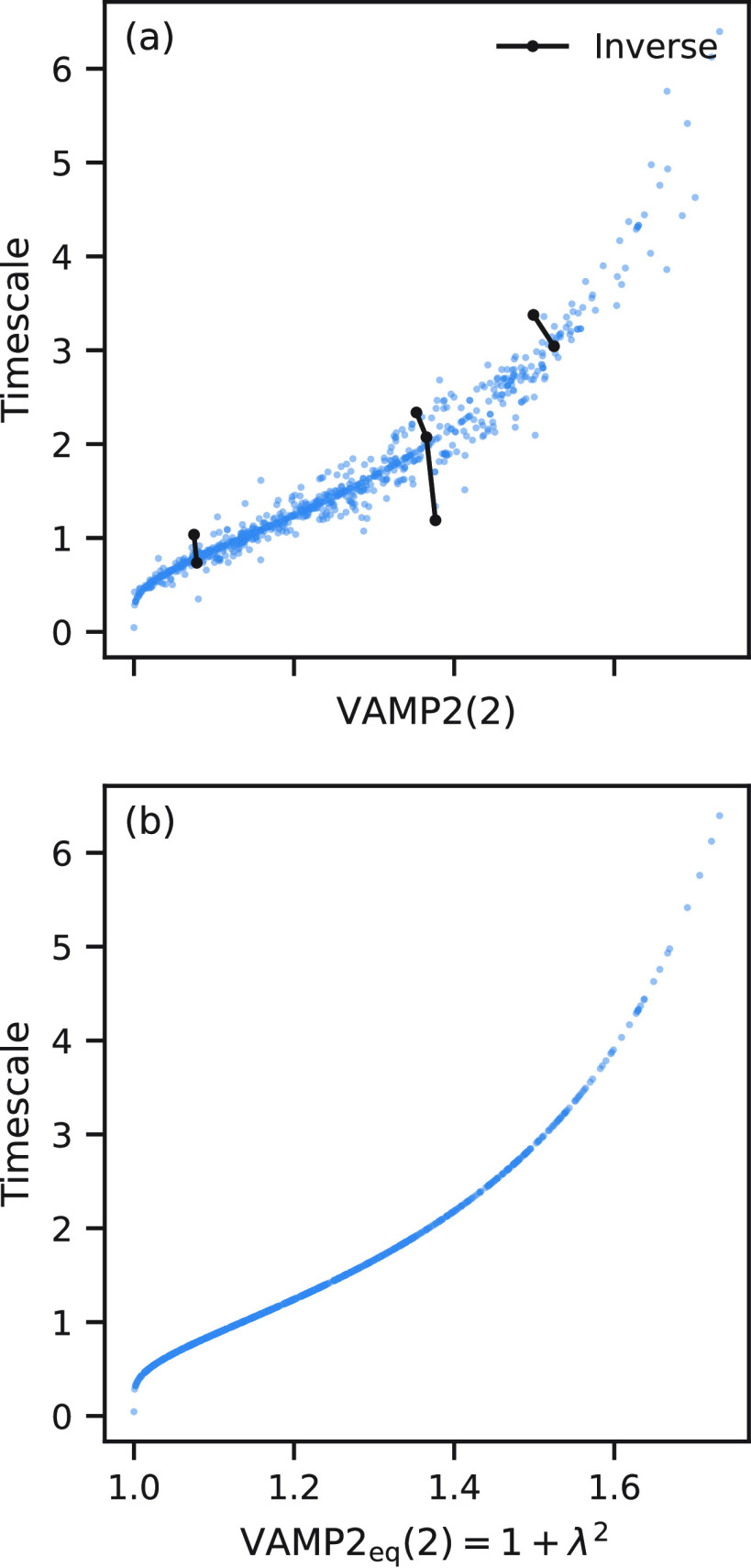
Relationship between implied time scales, *t*_2_, VAMP2(2), VAMP2_eq_(2) scores. Each of the 1000 blue points is calculated from a MSM estimated from a distinct simulated trajectory of 20 time steps. The trajectories were generated from the same three-state reference transition matrix (taken from Trendelkamp-Schroer et al.^22^). The estimated transition matrices were all estimated ensuring reversibility. (a)*t*_2_ as a function of VAMP2(2) scores and (b) *t*_2_ as a function of VAMP2_eq_(2). The black points labeled “Inverse” are example subsets of MSMs where the relationship between the implied time scale and VAMP2(2) score are inverted.

To ensure that this phenomenon was not an artifact of the processing pipeline, the effect was replicated with a three-state toy model (example 1 in Trendelkamp-Schroer et al.^22^). Here, 10,000 × 20-step trajectories were sampled from the same 3 × 3 transition matrix, and for each trajectory, count matrices (**C**_00/0*t*/*tt*_) were calculated. We assert that the differences in the count matrices arising from the finite sampling in this toy model are similar to the differences from different discretization schemes in the example of BBA. From each set of count matrices *t*_2_ and VAMP2(2) scores were estimated, and these are shown in Figure 3(a). Although *t*_2_ is clearly rank-correlated with VAMP2(2), the rank correlation is not perfect. Many subsets of these results form sets which are anticorrelated; three examples of this inverse relationship are shown as black lines labeled “Inverse”. These subsets mirror the effect seen in the BBA models in Figure 2. As a comparison, in panel (b), we plot the sum of the squares of the first two eigenvalues, VAMP2_eq_(2), which shows perfect rank correlation (as they must).

The reason for writing the VAMP2(*k*) score as the product of count matrices and eigenvectors/singular vector matrices is to facilitate data splitting in cross-validation. While we used bootstrapping for this work and thus mitigated this, the effect of data splitting would be to worsen the discrepancy between the count and transition matrices. This is because the count matrices are now estimated on different data compared to those of the eigenvector matrices.

Due to the problem of consistency between the matrices in eq 6 arising from (a) enforcing reversibility and (b) data splitting for cross-validation, we recommend that VAMP2(*k*) scores, either cross-validated or bootstrapped, should not be used for reversible and stationary MSMs. Instead, we recommend bootstrapping the sum of the squared eigenvalues (VAMP2_eq_(*k*)) directly from the reversible transition matrix. This has the same theoretical properties of the VAMP2(*k*) score (i.e., represents captured kinetic variance and link to variational theorem) while not (a) wasting data due to data splitting and (b) perfect correlation with the implied time scales. One may also consider other objectives, such as the sum of the first *k* implied time scales or eigenvalues, which can also be maximized via the variational principle.

### Eigenvectors May Change Definition with Change in Hyperparameters

**Figure 4:**
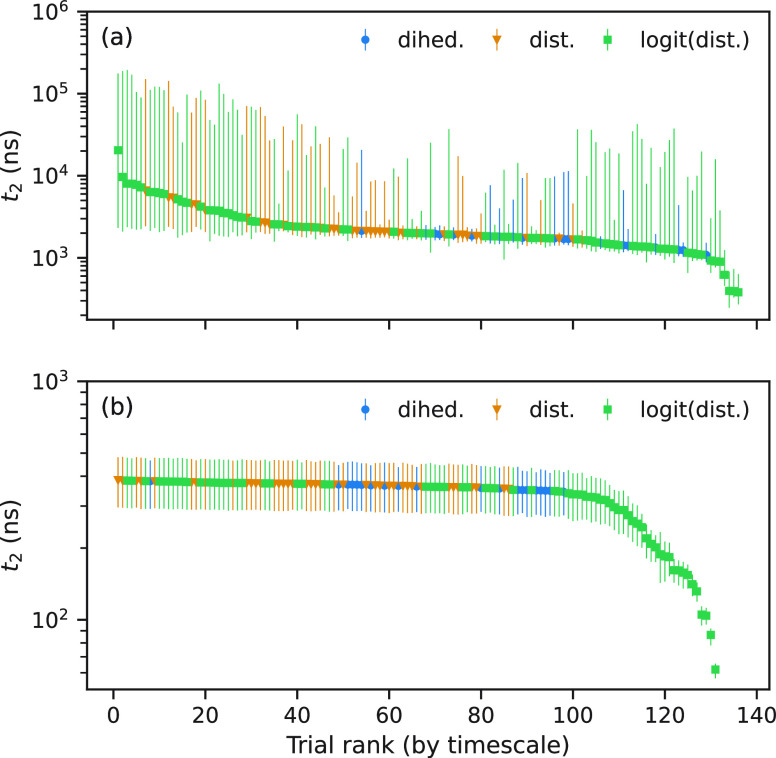
Time scales of randomly sampled hyperparameter trials. (a) BBA and (b) Chignolin. The vertical axis is the dominant time scale (*t*_2_); the horizontal axis is the trial rank. The solid disc and error bars are the median and 95% bootstrapped confidence intervals.

Figure 4 shows the results of the optimization through random selection: the distribution of the time scale (*t*_2_) of the dominant process (corresponding to the second right-eigenvector of the MSM transition matrix, ψ_2_) for each MSM of BBA (panel (a)) and Chignolin (panel (b)), ordered left to right with the highest value of *t*_2_ on the left. Each point is colored according to the feature used. According to previous research,^25^ we expect in both cases the dominant relaxation process to correlate with the folded-to-unfolded transition. We expect that the best model would be the one with the highest value of *t*_2_. However, implicit in this decision is that the implied time scale represents the same underlying relaxation process.

**Figure 5:**
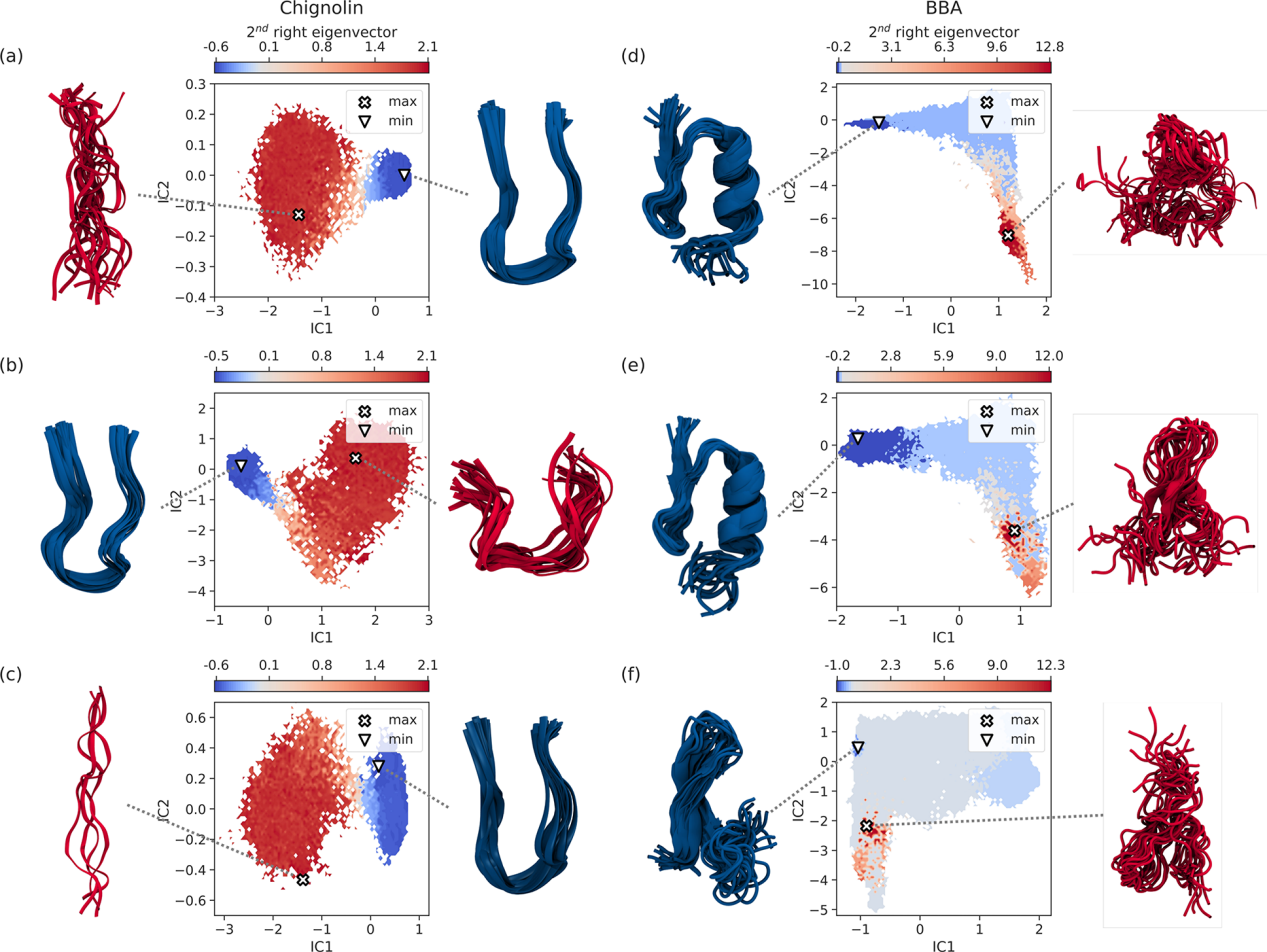
MSMs with different relaxation processes. Each panel shows ψ_2_ in the space of the first two tICA coordinates. Also shown is an ensemble of structures sampled from the microstates with the extreme values of ψ_2_. (a) Chignolin model 1, (the largest median *t*_2_ from random sampling). (b) Chignolin model 3 (the largest median *t*_2_ from Bayesian optimization of *t*_2_). (c) Chignolin model 4 (the largest *t*_2_ from Bayesian optimization of VAMP2_eq_(2) and VAMP2_eq_(2)/VAMP2_eq_(3)). (d) BBA model 1 (the largest median *t*_2_ from random sampling). (e) BBA model 2 (the second largest median *t*_2_ from random sampling). (f) BBA model 5 (the largest median *t*_2_ from Bayesian optimization of *t*_2_).

For Chignolin, both *t*_2_ and the corresponding relaxation process for different hyperparameters trials are similar, as can be seen by inspection of the models 1 and 2 (ranked 1 and 4, respectively, in Figure 4, see Table S1 and the Supporting Information for more detailed information on each model). In model 1, Figure 5(a) shows ψ_2_ as an unfolded–folded transition. In model 2, Figure S6(c) shows ψ_2_ as transitioning between a structure that is almost completely unfolded with only two non-native contacts and the folded state. The main difference between these models is the choice of feature: model 1 uses distances, while model 2 uses the logistic transform of distances (Figure S1, blue line). In contrast to the distances feature, this transform is less sensitive to changes in distances > 6Å, which may explain the slight difference in eigenvector definition.

In the case of BBA, the situation is different. The top two best performing models, models 1 and 2 (ranked 1 and 2, respectively, in Figure 4, see Table S1 and Supporting Information) show evidence of optimizing a similar relaxation mode, ψ_2_. Comparing models 1 and 2, we see a similar folded-unfolded transition (Figure 5(a) and (b)). Model 1 is more accurate, as the values of *t*_2_ show: 20.4 μs, 95%C.I. [2.3–176.2 μs] cf. 9.7 μs, 95%C.I. [2.1–188.7 μs]. This accords with differences in the hyperparameters: models 1 and 2 use the logistic distances feature, but model 1 has a logistic transform which is more sensitive to changes in contact distances between 0.1 Å —10 Å (see Figure S1) and more discrete basis functions (471 cf. 289, see Table S1) which can help resolve the dominant eigenvector more precisely.

However, the best-performing models with the other features have markedly different ψ_2_ values which do not correspond to the same transition as models 1 and 2. Model 3 is the best model with the distance feature (ranked 7 in Figure 4, see Table S1 and Supporting Information), and model 4 is the best model with the dihedrals feature (ranked 54 in Figure 4, and see Table S1 and Supporting Information). Both of these show markedly different transitions for ψ_2_, see Figures S21(c) and S24(c), respectively.

Thus, when optimizing MSMs the objective function (*t*_2_ in this case), ψ_2_ may change definition across the search space, and one is not comparing like-with-like when looking at *just* the objective function. This is consistent with the findings in Nagel et al.^52^ in which models with different processing methods had similar GMRQ scores, which then gave rise to different partitions of free energy space into metastable basins.

To mitigate this problem, we advocate checking the character of the eigenvectors when selecting appropriate hyperparameters to ensure that one is optimizing at least a consistent set of relaxation processes.

### Bayesian Optimization May Optimize Different Processes

We tested whether Bayesian optimization could increase the *t*_2_ by selecting better hyperparameters. We optimized the search space in Table 2 using both single-objective and dual-objective optimization, with objectives based on the time scales, *t*_2/3_, and the VAMP2_eq_(2/3) scores, see Table 3 for a list of optimization experiments. The optimization using dual objectives of *t*_2_ with *t*_2_/*t*_3_ (and the VAMP2_eq_ equivalent) was prompted by the observation that from the randomly sampled hyperparameter trial data set, there were many models with similar values of *t*_2_ but with a wide range of time scale gaps *t*_2_/*t*_3_. A large time scale gap gives rise to models that are more accurate when truncated and coarse-grained into a two-state model. Our hope was therefore to bias the optimization results in favor of both large *t*_2_ and a large separation of time scales. The optimization trajectories, which show the largest value of *t*_2_ (vertical axis) in all trials up to the current trial number (horizontal axis) are shown in Figure S32.

Single objective optimization of both *t*_2_ and VAMP2_eq_(2) increased *t*_2_ for Chignolin and BBA. For Chignolin, the increase was modest, between 2.4%—5.5% for all four objective functions. The single objective optimization of *t*_2_ had the smallest increase (Figure S32(a), red squares) while the multiobjective optimization of the VAMP2_eq_(2) and VAMP2_eq_(2)/VAMP2_eq_(3) gave the largest increase in *t*_2_ (Figure S32(c), blue squares), although the increase in the gap was modest (see Figure S33(c)).

The small *t*_2_ increase for Chignolin is unsurprising given the consistency of *t*_2_ across the randomly sampled hyperparameter trials. However, the *t*_2_ optimized MSM, model 3 (see Table S1 and Supporting Information) shows a partially folded to folded transition in Figure 5(b) rather than the fully unfolded to folded transition in model 1 (Figure 5(b)). In terms of the values of the hyperparameters, the optimization has changed the tICA lag time significantly (from 71 ns in model 1 to 3 ns in model 3; the other hyperparameters have remained largely unchanged). The VAMP2_eq_(2) and VAMP2_eq_(2)/VAMP2_eq_(3) optimized MSM, model 4 (see Table S1 and Supporting Information), shows the same unfolded–folded transition as model 1 (see Figure 5(c). Both the dual-objective optimizations (*t*_2_ with *t*_2_/*t*_3_ and the VAMP equivalent) increased both the *t*_2_ and the separation of time scales (see Figure S33).

For BBA, the single objective optimization of *t*_2_ and VAMP2_eq_(2) increased *t*_2_ by 128% and 135%, respectively. However, these models have not optimized the same relaxation process as the incumbent from the randomly sampled hyperparameters, model 1. The *t*_2_ optimized MSM, model 5, (see Table S1 and Supporting Information), denotes a transition between two misfolded structures (see Figure 5(f). This is perhaps surprising given that the main difference between the two model specifications is that change in the logistic transform (see Figure S1 for the difference between model 1’s and model 5’s logistic transform). In contrast, the VAMP2_eq_(2) optimized model shows a transition similar to those of models 1 and 2 (see Figure S30(c)). Both of the dual objective optimization runs did not improve *t*_2_ significantly, although, in the case of optimization of *t*_2_ with *t*_2_/*t*_3_, the gap increased significantly (see Figure S33(b)).

The implications for Bayesian optimization are similar to the lessons learned from random optimization: changing hyperparameters can change the optimized process, meaning that one must analyze the character of the eigenvectors to ensure one is optimizing the same processes.

### Lag Time and Number of Scored Eigenvectors Do Not Affect Model Selection

When evaluating MSMs using a variational score, one must specify both the Markov lag time (τ) and the number of eigenvectors to score (*k*). However, both these choices affect the VAMP score, although it is not clear whether these choices affect the model ranking. To test how these choices affect model selection, we measured the consistency in model rank for BBA, as measured by the VAMP2_eq_(k), using the Spearman’s rank correlation coefficient at (a) different lag times for given values of *k* and (b) at different values of *k* at a given lag time.

**Figure 6:**
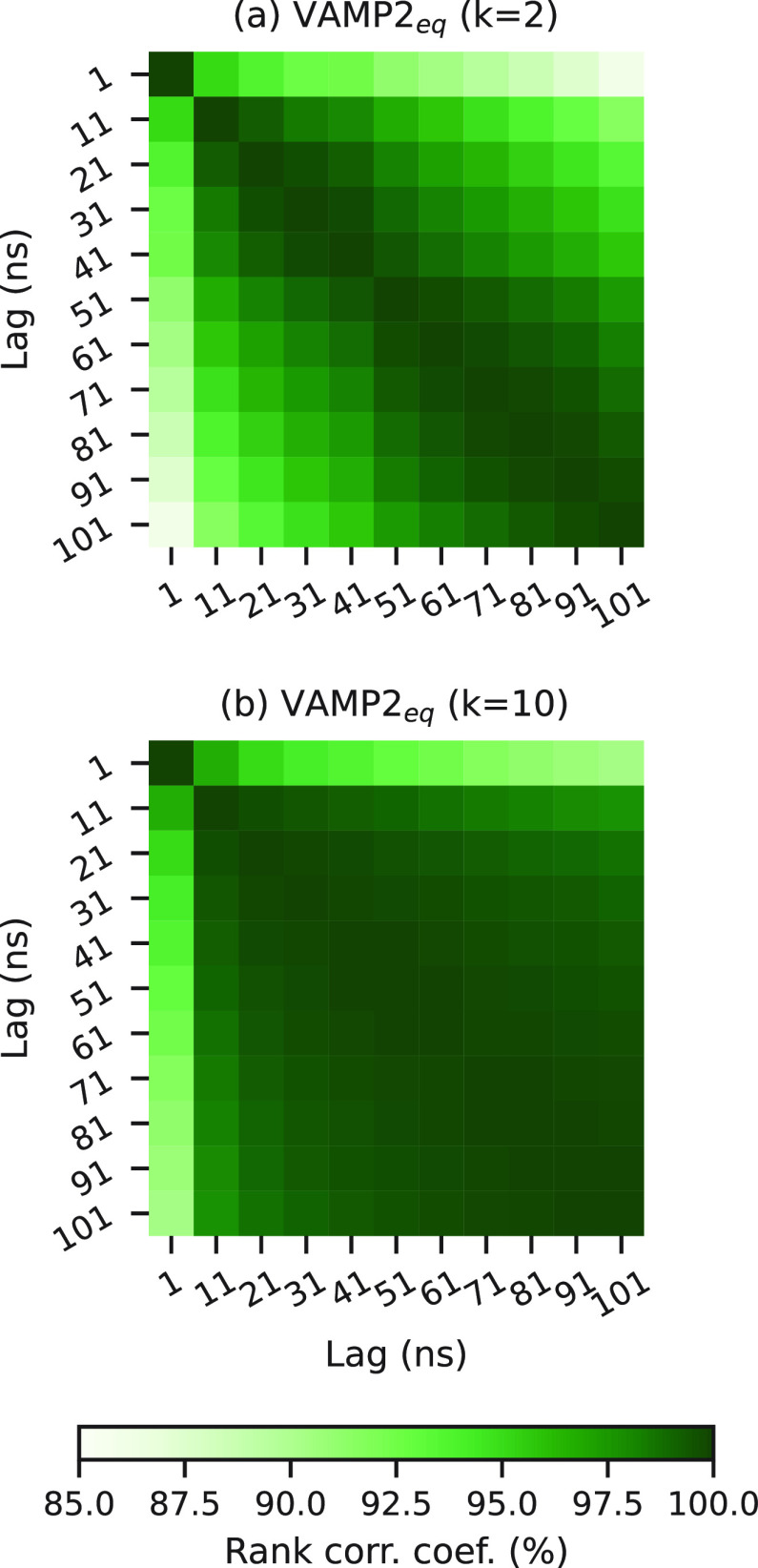
Consistency of VAMP2_eq_(*k*) rank with Markov lag time, τ for BBA. The *i*, *j*th cell in (a) shows the Spearman’s rank correlation coefficient of VAMP2_eq_(2) for each trial measured at the *i*th lag time, with VAMP2_eq_(2) measured at the *j*th lag time. (b) Same measurements with VAMP2_eq_(10) score, respectively.

Figure 6 shows the consistency between BBA model rankings at different lag times (1 ns ≤ τ ≤ 101 ns) with *k* = 2 (panel (a)) and with *k* = 10 (panel (b)). In addition, scatter plots of the data used to calculate these coefficients for *k* = 2, 3, 5, and 10 are shown in Figures S33–S37. Across all lags and for both small (*k* = 2) and large (*k* = 10) numbers of scored eigenvectors, the consistency in the model ranking is high (greater than 85%). The consistency between models with lag times τ > 1 ns is much greater, with rank correlations up to 100%. This effect is most pronounced for *k* = 10 scored eigenvectors. In particular, good consistency is achieved at lag times smaller than those required for the model to be Markovian (τ = 41 ns).

**Figure 7:**
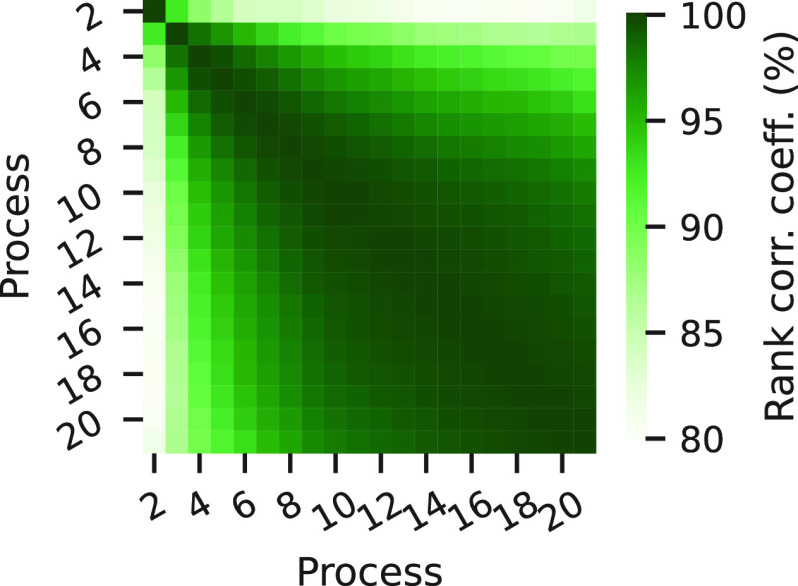
Consistency of VAMP2_eq_(*k*) rank with number of scored eigenvectors for BBA. The ranks of trials in the row *k* are compared to their rank at the column *k* using the Spearman’s rank correlation coefficient at a lag time of 41 ns.

Figure 7 shows the consistency between model rankings at different numbers of scored eigenvectors (2 ≤ *k* ≤ 21) at a lag time of 41 ns (the value used in all previous analyses for BBA). Again, the consistency is generally high with a rank correlation between all pairs of *k* of at least 80%. The ranking is most consistent between values of *k* larger than 4. From these two analyses taken together, we see that for long lag times and a large number of scored eigenvectors, model ranking is significantly affected by the choice of τ and *k*.

## Conclusions

This work has drawn a complex picture of MSM optimization which suggests that model selection of MSM hyperparameters by inspection of a single objective measure (e.g., *t*_2_) is not advisible as changes in hyperparameters can change the physical meaning of the MSM eigenvectors. The commonly used VAMP2 metric when used with the assumption of reversibility can give rise to rankings of MSMs which are inconsistent with the implied time scales. We suggest that this is due to a numerical, rather than a theoretical, problem. In its place, we suggest using the sum of the square eigenvalues or other model observables, e.g., *t*_2_, with bootstrapping to estimate uncertainty.

Bayesian optimization of MSM hyperparameters is possible using Parzen estimators for the surrogate function. TPEs can easily model the different types of hyperparameters (continuous, integer, and discrete) and improve the implied time scales. Multiobjective optimization can be used but does not give a clear advantage overoptimizing a single objective. Caution must again be exercised, as this can give rise to models in which the meaning of the eigenvectors can change.

We also showed that selecting lag times and several scored eigenvectors in the objective function does not drastically change the ranking of the hyperparameters, as long as both are sufficiently large. However, we emphasize the importance of choosing a lag time that is both sufficiently small to resolve interesting processes and large enough to meet the model assumption of Markovianity.

While we have explored a number of different hyperparameters in this work, one cannot hope to cover the full range of modeling approaches such as different dimensionality reduction methods (e.g., PCA,^28^ RMA^29^ etc.), clustering (e.g., k-hybrids,^65^ Ward clustering,^51^ density based clustering^28^ etc.), and coarse graining of the MSM (e.g., hidden Markov models,^66^ the Hummer–Szabo method,^67^ variationally optimizing the coarse-grained states based on the Kemeny constant,^68^ identification of Markovian transition states,^69^ along with many others^70−72^). Together they affect the structural resolution of the metastable states and thus the mechanism and coarse-grained kinetic parameters. In addition, machine learning methods, such as VAMPnets^49^ which replace many of the processing steps described here, introduce their own modeling choices (e.g., model architecture, learning hyperparameters) which should be explored.

Taken together, these observations suggest several recommendations:

1. Randomly sample a range of hyperparameters and use the VAMP2_eq_ or *t*_2_ (or the time scale of interest) to rank hyperparameters. Make sure to include many different modeling approaches.
2. Use a small subset of models with different lag times (τ) and score with a range of eigenvectors (*k*) and choose τ and *k* such that VAMP2_eq_ is independent of both.
3. Inspect eigenvectors to check for consistency across different hyperparameters.
4. Bayesian optimization of *t*_2_ or VAMP2_eq_ can be used to optimize hyperparameters but the eigenvectors must be inspected for consistency.
5. Along with model observables such as implied time scales, the structural resolution of the metastable states and the implied mechanisms must also be checked for interpretability and consistency.

## Data Availability

The molecular dynamics trajectories used in this work were used with permission from D. E. Shaw Research. All processing and analysis scripts for the experiments carried out and instructions on how to reproduce this work can be found at www.github.com/RobertArbon/msm_sensitivity and www.github.com/RobertArbon/msm_sensitivity_analysis.

## Supporting Information Available

37 additional figures, summary of all MSMs built for this study, analysis of both BBA and Chignolin models, and results for the optimization carried out (PDF)
