# Supplementary material for: Markov State Models: To Optimize or Not to Optimize
Source: J Chem Theory Comput. 2024 Jan 2;20(2):977–88. doi: 10.1021/acs.jctc.3c01134 (PMC10809420; doi:10.1021/acs.jctc.3c01134)
Supplement: Supplementary file 1 — ct3c01134_si_001.pdf [file ct3c01134_si_001.pdf]

Supporting Information
Markov state models: to optimize or not to optimize

Robert E. Arbon,[†,‡] Yanchen Zhu,[†] and Antonia S.J.S. Mey[*,†]

†*EaStCHEM School of Chemistry, David Brewster Road, Joseph Black Building, The King's Buildings, Edinburgh, EH9 3FJ, UK*

‡*Redesign Science, 180 Varick St, New York, NY 10014, USA*

E-mail: antonia.mey@ed.ac.uk

Model summaries

Table 1: MODEL SUMMARIES. A summary of the models discussed in the main text. The logistic transform of contact distances is specified below in terms of the center, c, and steepness, s, as: 'logit(dist.)' with '(c, s)' underneath, in units of Å and $Å^{-1}$

Protein	No.	Lag time (ns)	Feature	# cluster centers	TICA lag (ns)	TICA dim.	Optimisation method	# initialization trials	# optimisation trials	Objective	t_2 (µs)
Chignolin	1	31	dist.	488	71	15	Random	NA	140	t_2	0.379 (0.296, 0.470)
Chignolin	2	31	logit(dist.) (2.2, 0.61)	471	60	20	Random	NA	140	t_2	0.377 (0.294, 0.466)
Chignolin	3	31	dist.	469	3	15	Bayesian	131	100	t_2	0.383 (0.316, 0.463)
Chignolin	4	31	dist.	986	15	18	Bayesian	55	150	$V_{eq}(2) + V_{eq}(2)/V_{eq}(3)$	0.396 (0.326, 0.477)
BBA	1	41	logit(dist.) (2.2, 0.61)	471	60	20	Random	NA	140	t_2	20.4 (2.3, 176.2)
BBA	2	41	logit(dist.) (8.0, 2.9)	289	67	18	Random	NA	140	t_2	9.7 (2.1, 188.7)
BBA	3	41	dist.	485	52	19	Random	NA	140	t_2	6.6 (2.4, 150.4)
BBA	4	41	dihed.	471	99	13	Random	NA	140	t_2	2.1 (1.8, 20.6)
BBA	5	41	logit(dist.,) (7.96, 0.33)	444	53	20	Bayesian	136	100	t_2	42.8 (4.7, 286.3)
BBA	6	41	logit(dist.,) (7.7, 0.74)	957	71	15	Bayesian	136	100	$V_{eq}(2)$	54.2 (4.0, 448.2)

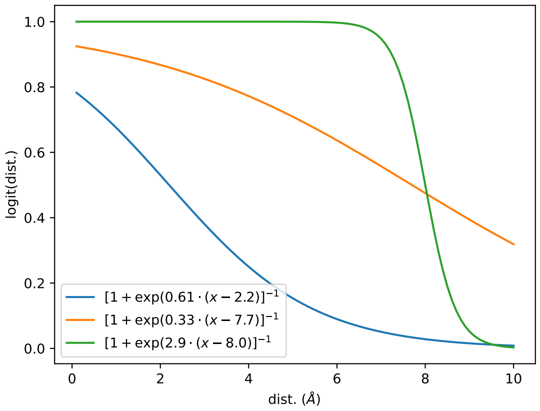

Figure 1: Logistic transforms in select models. The horizontal axis is the contact distance in Å, the vertical axis shows the logistic transform of that distance for BBA model 1 (blue), model 4 (orange) and model 5 (green).

Detailed model summaries

Chignolin model 1

This model has the largest median t_2 after random sampling optimisation.

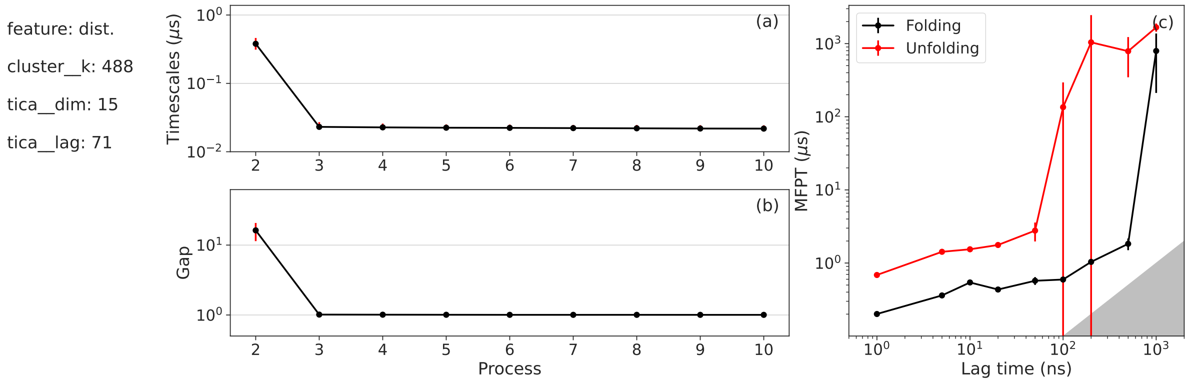

Figure 2: Chignolin, model 1 timescales. Text inset shows the MSM hyperparameters; panel (a) shows the implied timescales for the first nine slow relaxation processes ($\tau = -1$ ns); panel (b) shows the gap between successive successive timescales: the gap for process i is defined as t_i/t_{i+1} ($\tau = -1$ ns); panel (c) shows the mean first passage time between the the unfolded and folded state as a function of τ.

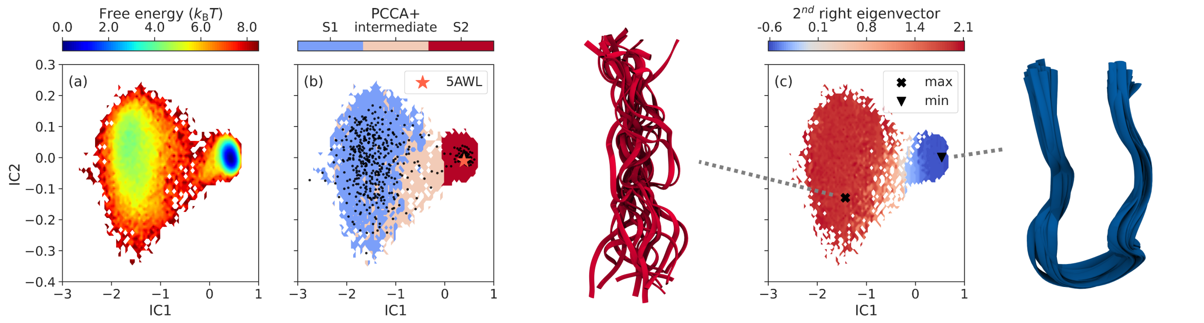

Figure 3: Chignolin, model 1 free-energy surface. Each panel shows different quantities projected onto the first two TICA components (IC1, IC2). Panel (a) shows the free energy surface; panel (b) shows the PCCA+ clustering into folded, unfolded and intermediate states with the crystal structure (PDB accession code 5AWL) marked with a star; panel (c) shows the 2nd right eigenvector (which corresponds to the slowest relaxation process) with an ensemble of structures corresponding to the extremes values of the eigenvector ('min', 'max' marked with a triangle and cross respectively).

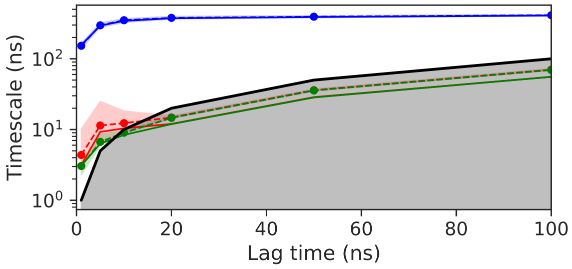

Figure 4: Chignolin, model 1 validation. The implied timescales plotted as a function of the lag-time. Blue, red, and green lines correspond to the slowest, second slowest, and third slowest process respectively. The solid lines correspond to the timescales from the maximum likelihood MSM. The dashed lines correspond to the mean of Bayesian MSMs. The coloured regions refer to the 0.95 confidence interval. The shaded region shows when the Markov lag time becomes equal to or longer than the implied timescale.

Chignolin model 2

This model has the largest median t_2 using the logistic distance feature from random sampling.

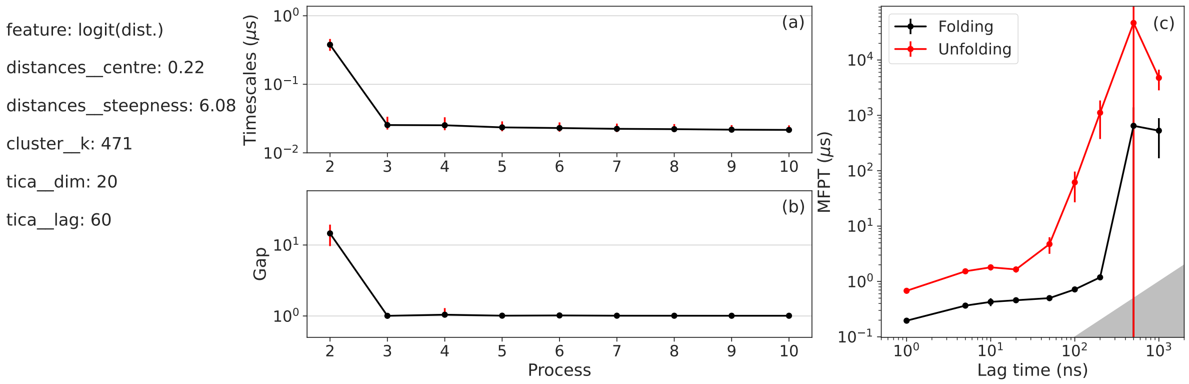

Figure 5: Chignolin, model 2 timescales. See the caption for figure 2.

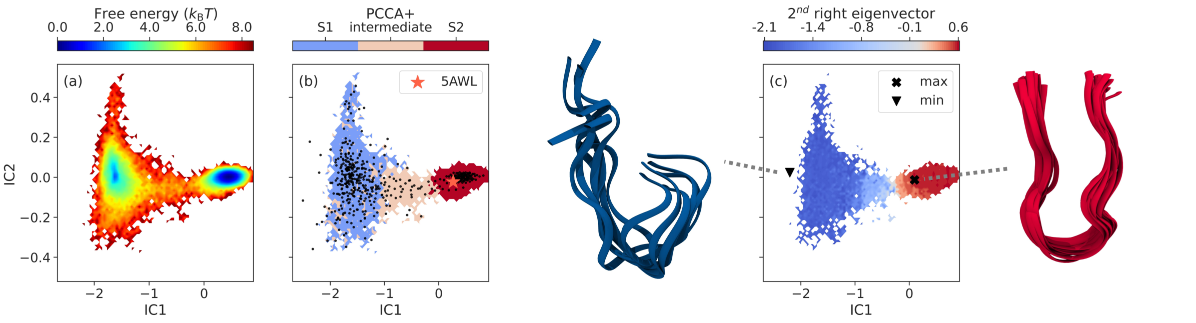

Figure 6: Chignolin, model 2 free energy surface. See the caption for figure 3.

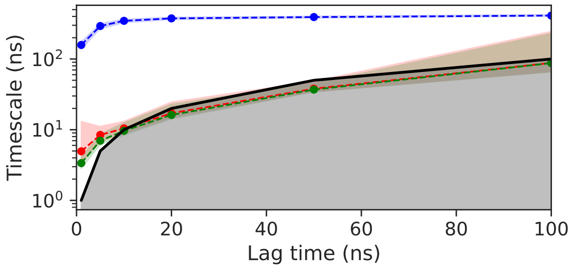

Figure 7: Chignolin, model 2 validation. See the caption for figure 4.

Chignolin model 3

This model has the largest median t_2 after Bayesian optimisation of t_2.

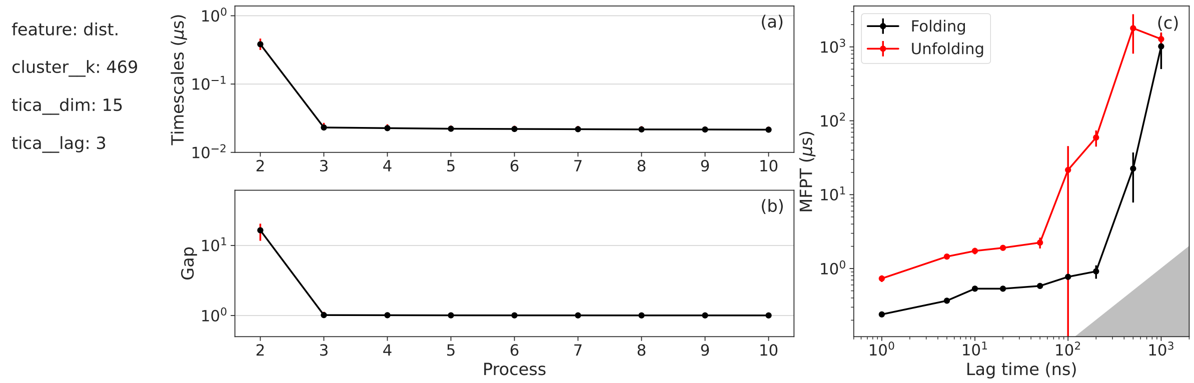

Figure 8: CHIGNOLIN, MODEL 3 TIMESCALES. See the caption for figure 2.

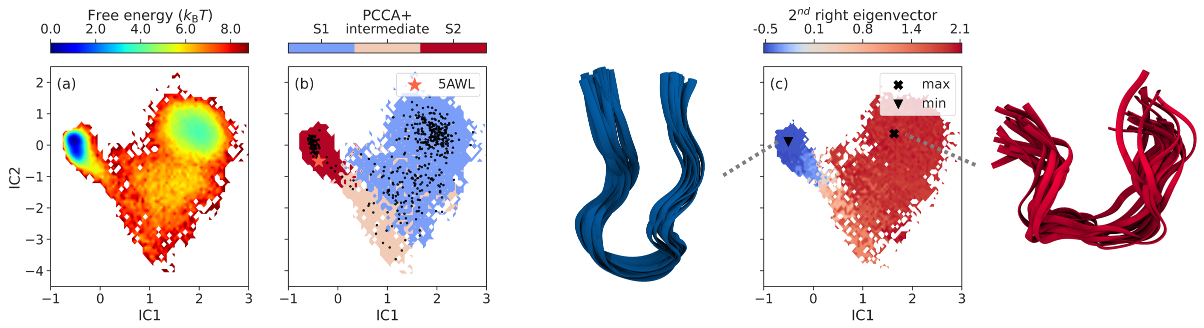

Figure 9: CHIGNOLIN, MODEL 3 FREE ENERGY SURFACE. See the caption for figure 3.

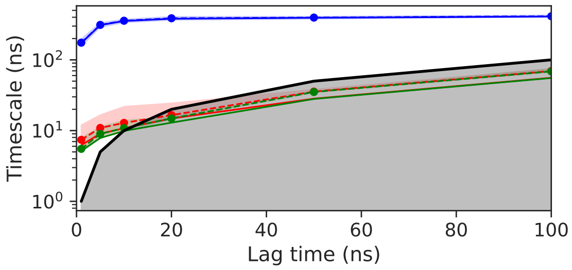

Figure 10: CHIGNOLIN, MODEL 3 VALIDATION. See the caption for figure 4.

Chignolin model 4

This model has the largest median t_2 after Bayesian optimisation of $\text{VAMP2}_{eq}(2)$ and $\text{VAMP2}_{eq}(2)/\text{VAMP2}_{eq}(3)$.

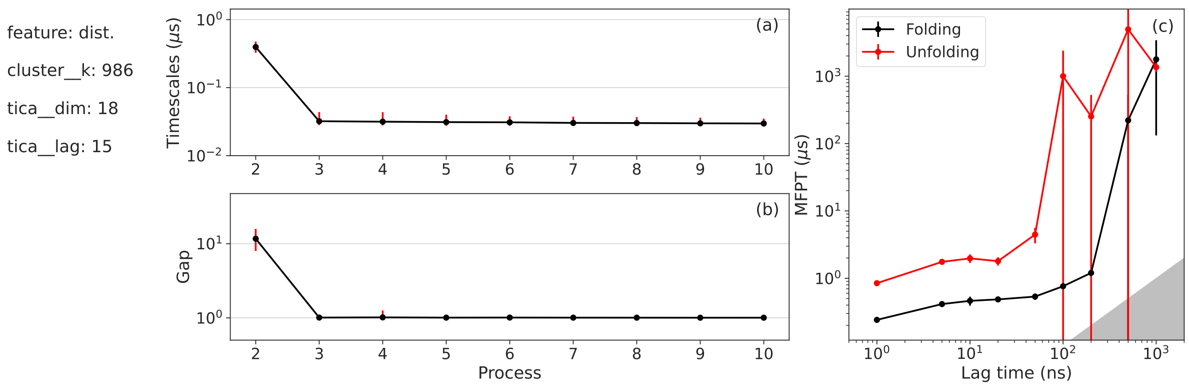

Figure 11: Chignolin, model 4 timescales. See the caption for figure 2.

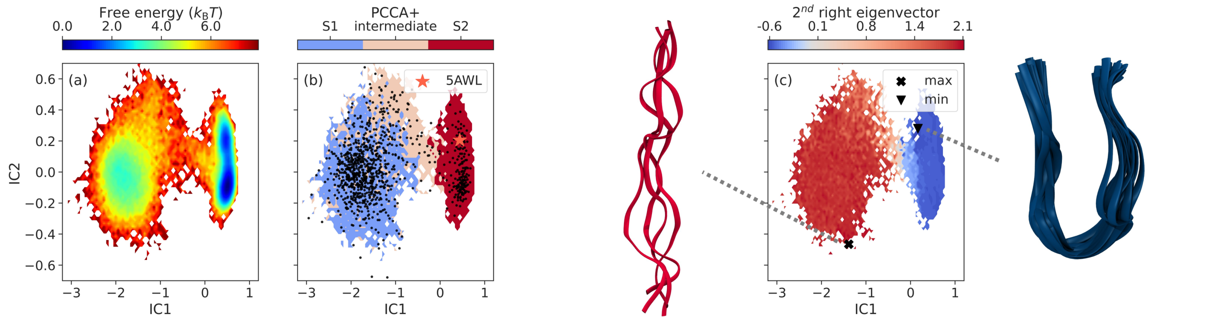

Figure 12: Chignolin, model 4 free energy surface. See the caption for figure 3.

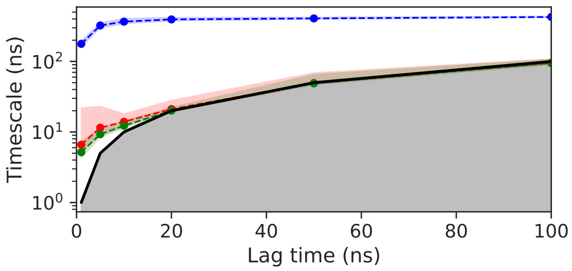

Figure 13: Chignolin, model 4 validation. See the caption for figure 4.

BBA model 1

This model has the largest median t_2 after random sampling.

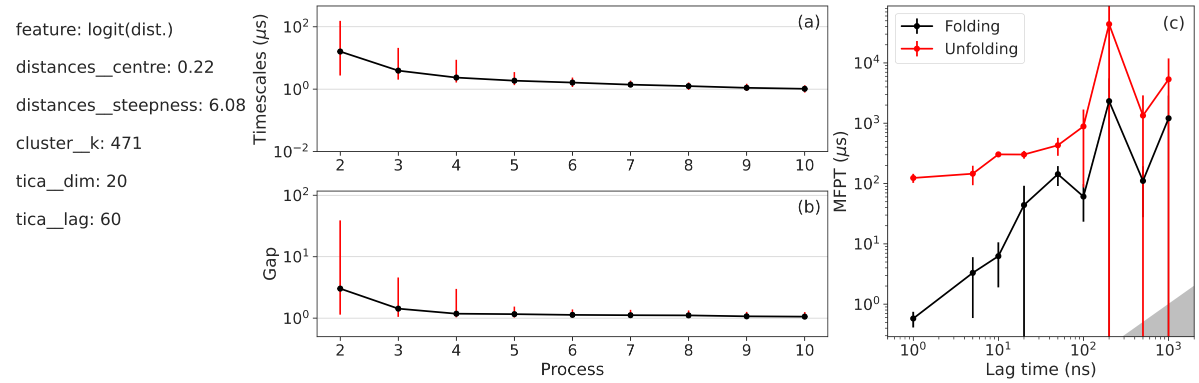

Figure 14: BBA, MODEL 1 TIMESCALES. Text inset shows the MSM hyperparameters; panel (a) shows the implied timescales for the first nine slow relaxation processes ($\tau = 41\,\text{ns}$); panel (b) shows the gap between successive successive timescales: the gap for process i is defined as t_i/t_{i+1} ($\tau = 41\,\text{ns}$); panel (c) shows the mean first passage time between the the unfolded and folded state as a function of τ.

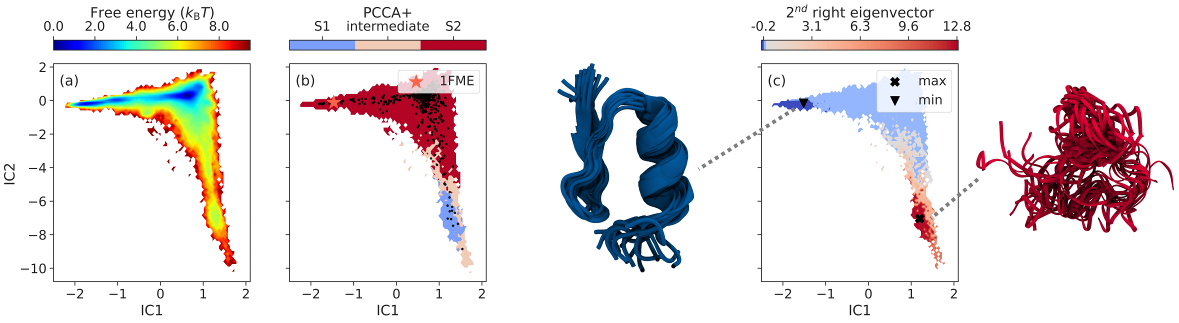

Figure 15: BBA, MODEL 1 FREE ENERGY SURFACE. Each panel shows different quantities projected onto the first two TICA components (IC1, IC2). Panel (a) shows the free energy surface; panel (b) shows the PCCA+ clustering into folded, unfolded and intermediate states with the crystal structure (PDB accession code 1FME) marked with a star; panel (c) shows the 2nd right eigenvector (which corresponds to the slowest relaxation process) with an ensemble of structures corresponding to the extremes values of the eigenvector ('min', 'max' marked with a triangle and cross respectively).

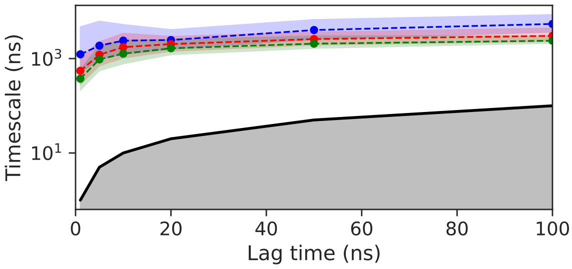

Figure 16: BBA, MODEL 1 VALIDATION. The implied timescales plotted as a function of the lag-time. Blue, red, and green lines correspond to the slowest, second slowest, and third slowest process respectively. The solid lines correspond to the timescales from the maximum likelihood MSM. The dashed lines correspond to the mean of Bayesian MSMs. The coloured regions refer to the 0.95 confidence interval. The shaded region shows when the Markov lag time becomes equal to or longer than the implied timescale.

BBA model 2

This model has the second largest median t_2 after random sampling.

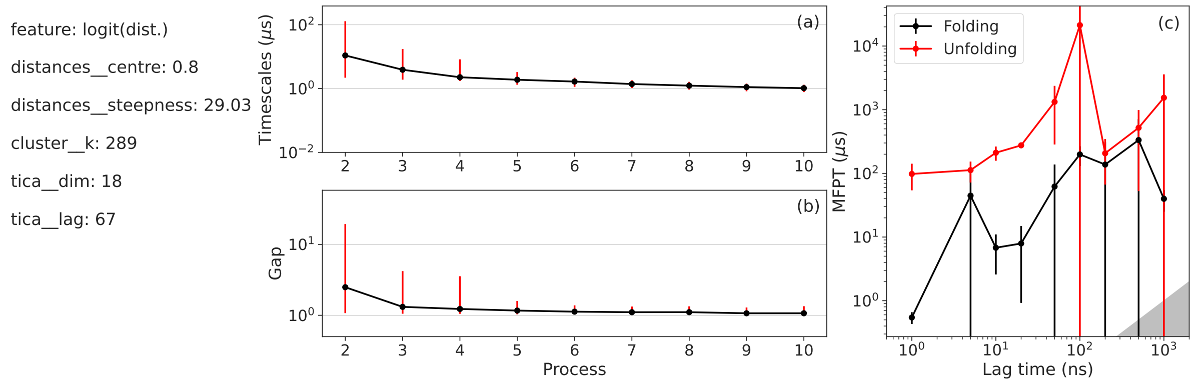

Figure 17: BBA, MODEL 2 TIMESCALES. See the caption for figure 14.

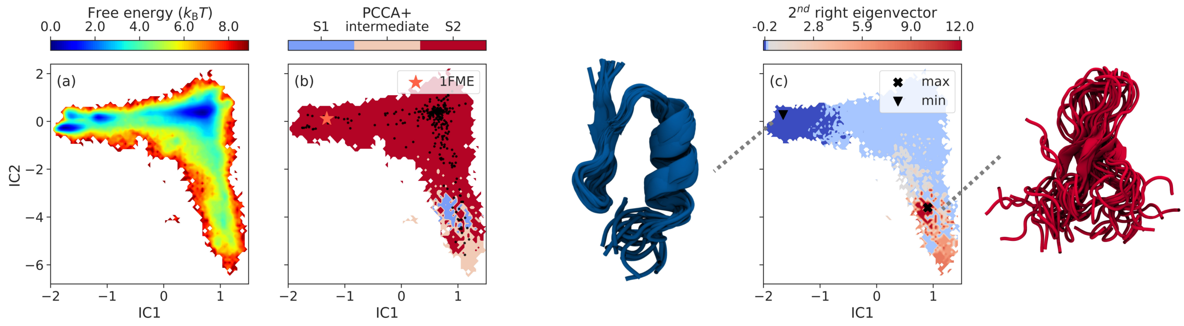

Figure 18: BBA, MODEL 2 FREE ENERGY SURFACE. See the caption for figure 15.

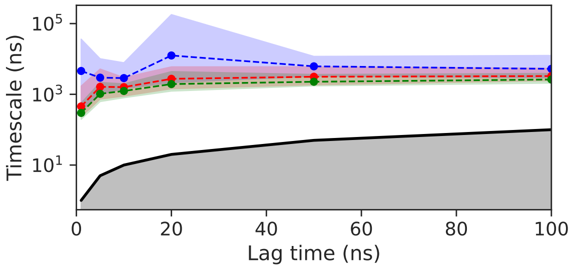

Figure 19: BBA, MODEL 2 VALIDATION. See the caption for figure 16.

BBA model 3

This model has the largest median t_2 using the distance feature after random sampling.

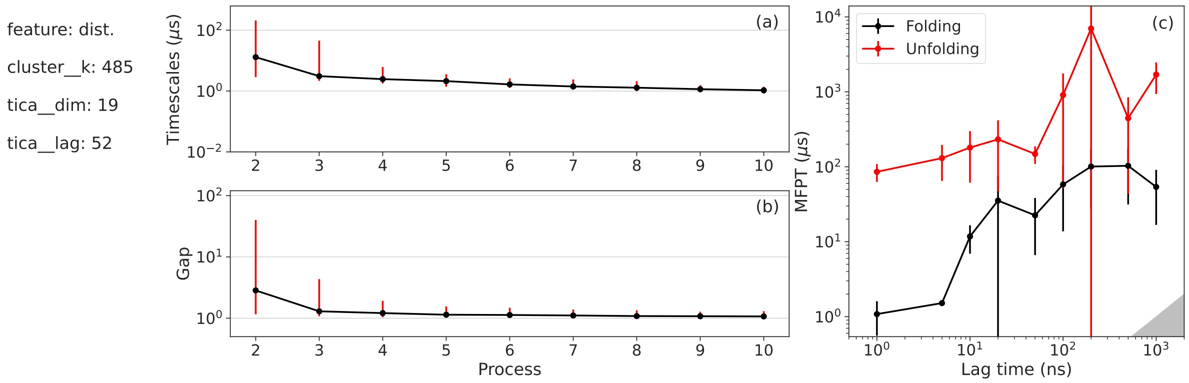

Figure 20: BBA, MODEL 3 TIMESCALES. See the caption for figure 14.

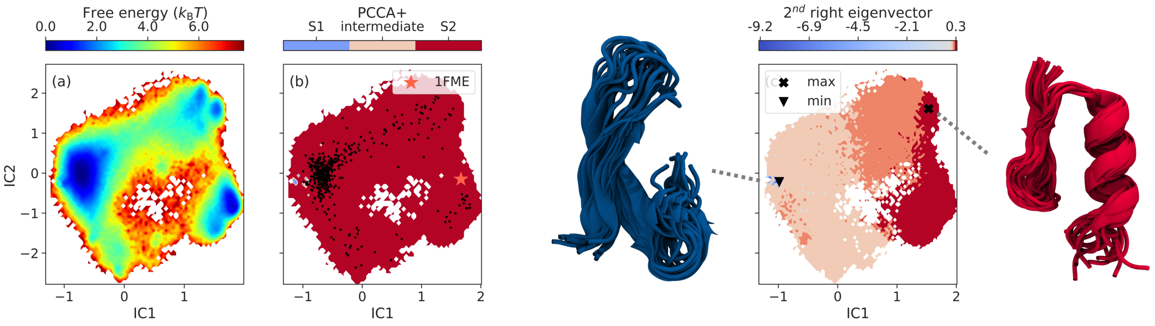

Figure 21: BBA, MODEL 3 FREE ENERGY SURFACE. See the caption for figure 15.

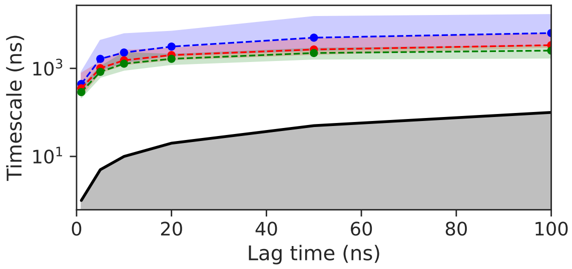

Figure 22: BBA, MODEL 3 VALIDATION. See the caption for figure 16.

BBA model 4

This model has the largest median t_2 using the dihedral feature after random sampling.

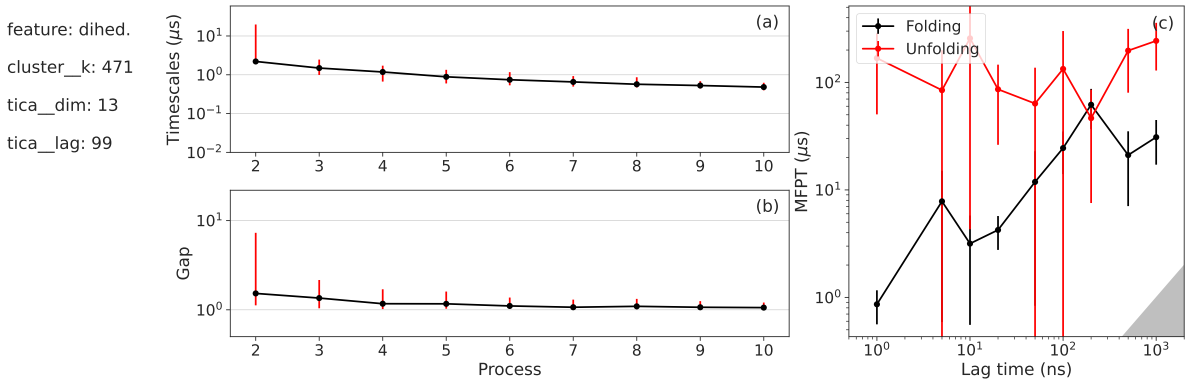

Figure 23: BBA, MODEL 4 TIMESCALES. See the caption for figure 14.

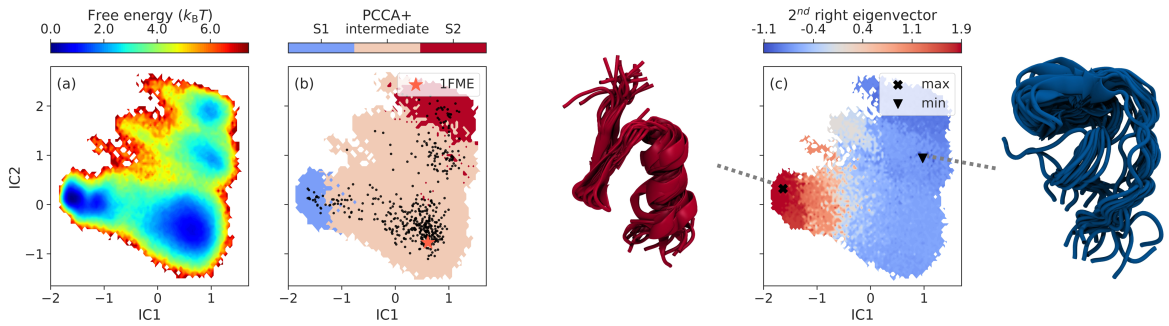

Figure 24: BBA, MODEL 4 FREE ENERGY SURFACE. See the caption for figure 15.

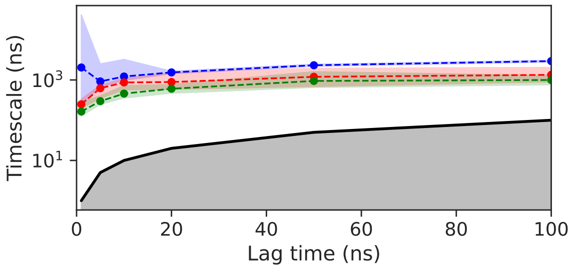

Figure 25: BBA, MODEL 4 VALIDATION. See the caption for figure 16.

BBA model 5

This model has the largest median t_2 after Bayesian optimisation of t_2.

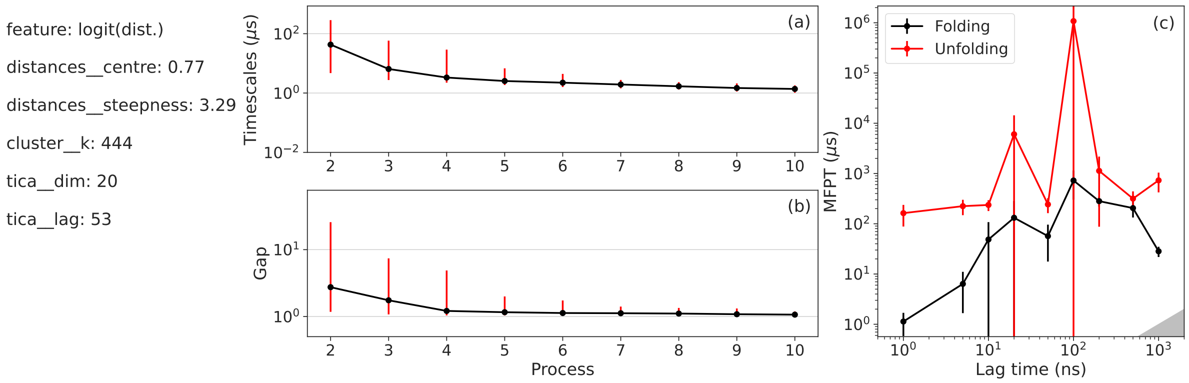

Figure 26: BBA, MODEL 5 TIMESCALES. See the caption for figure 14.

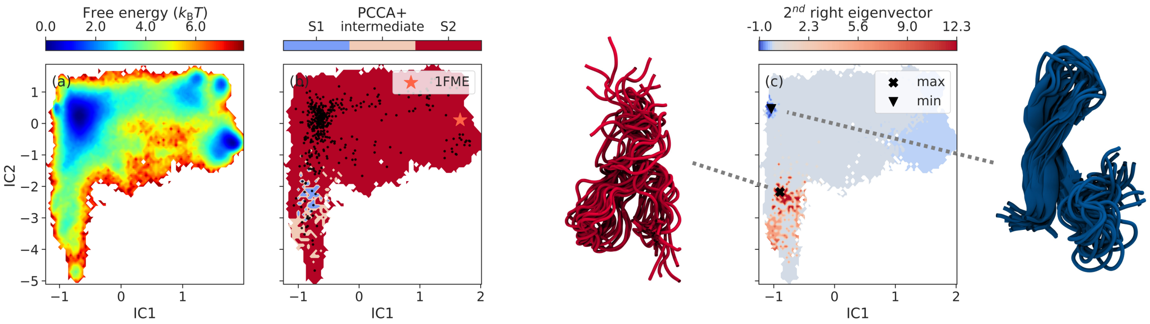

Figure 27: BBA, MODEL 5 FREE ENERGY SURFACE. See the caption for figure 15.

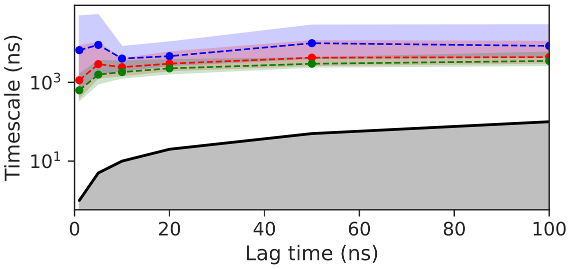

Figure 28: BBA, MODEL 5 VALIDATION. See the caption for figure 16.

BBA model 6

This model has the largest median t_2 after Bayesian optimisation of VAMP2$_{eq}$(2).

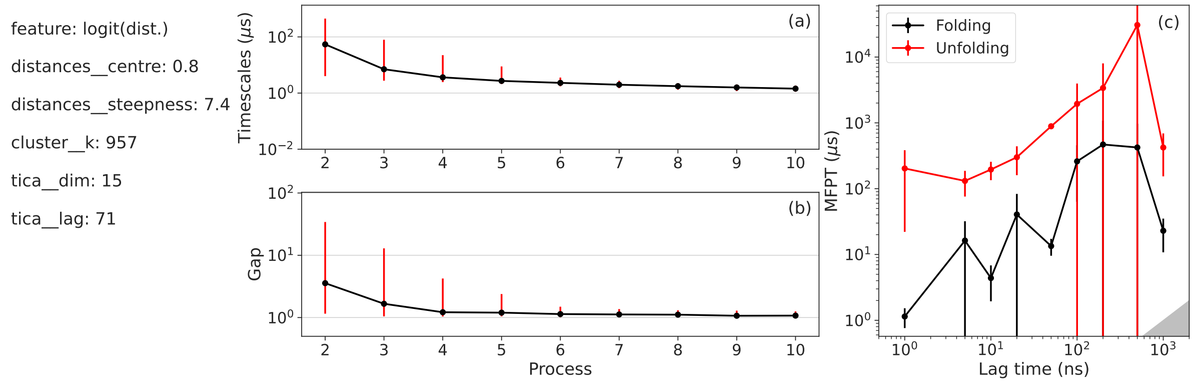

Figure 29: BBA, MODEL 6 TIMESCALES. See the caption for figure 14.

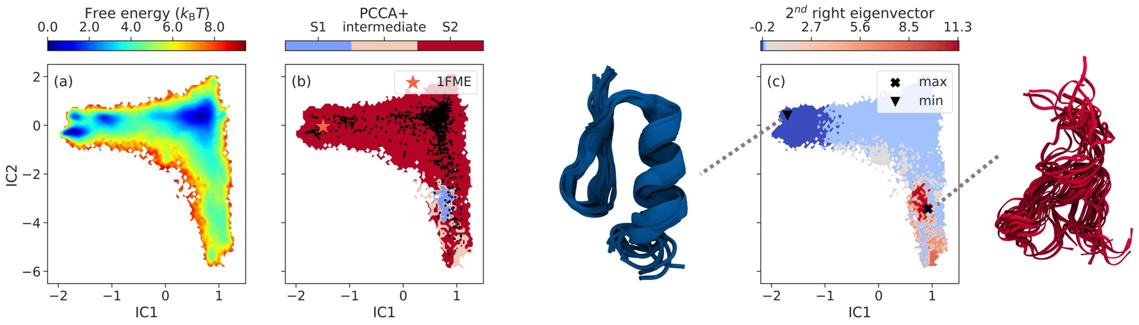

Figure 30: BBA, MODEL 6 FREE ENERGY SURFACE. See the caption for figure 15.

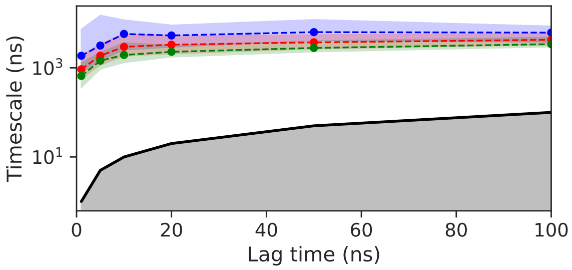

Figure 31: BBA, MODEL 6 VALIDATION. See the caption for figure 16.

Multi-objective optimisation

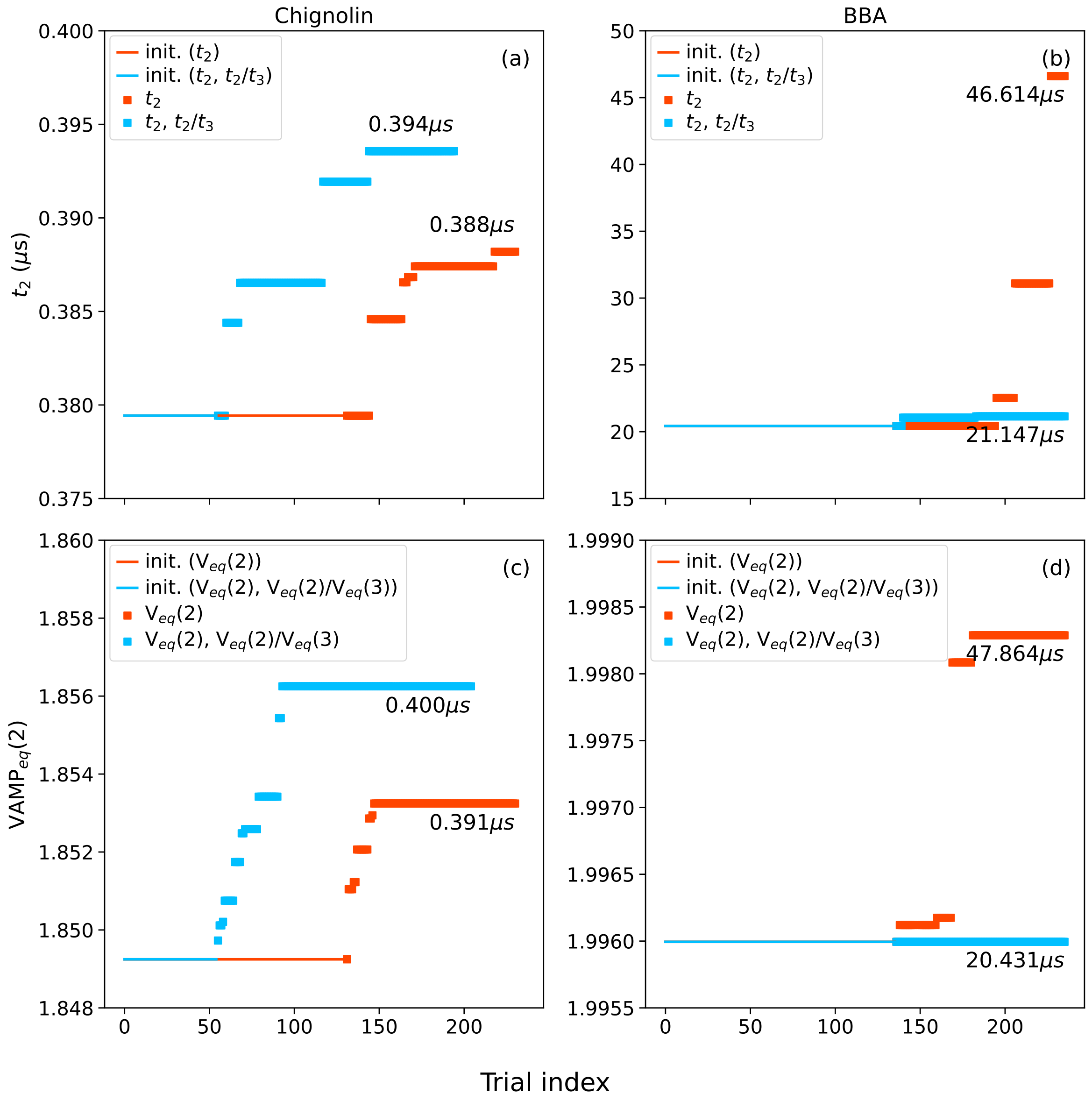

Figure 32: Optimization of MSMs of Chignolin and BBA. The vertical axis is the optimization objective, the horizontal axis is the trial number. The thin line refers to the incumbent over the initialization data ('init.'), and the squares are the incumbent at each trial number. Panels (a) and (c) refer to Chignolin, (b) and (d) to BBA. Panels (a) and (b) refer to Bayesian optimization with either t_2 (red), or dual-objective optimization of both t_2 and the timescale gap, t_2/t_3. Panels (c) and (d) refer to optimization of VAMP2$_{eq}(2)$ (red) and dual-objective optimization of both VAMP2$_{eq}(2)$ and VAMP2$_{eq}(2)$/VAMP2$_{eq}(3)$. The optimized values of t_2 are shown as labels.

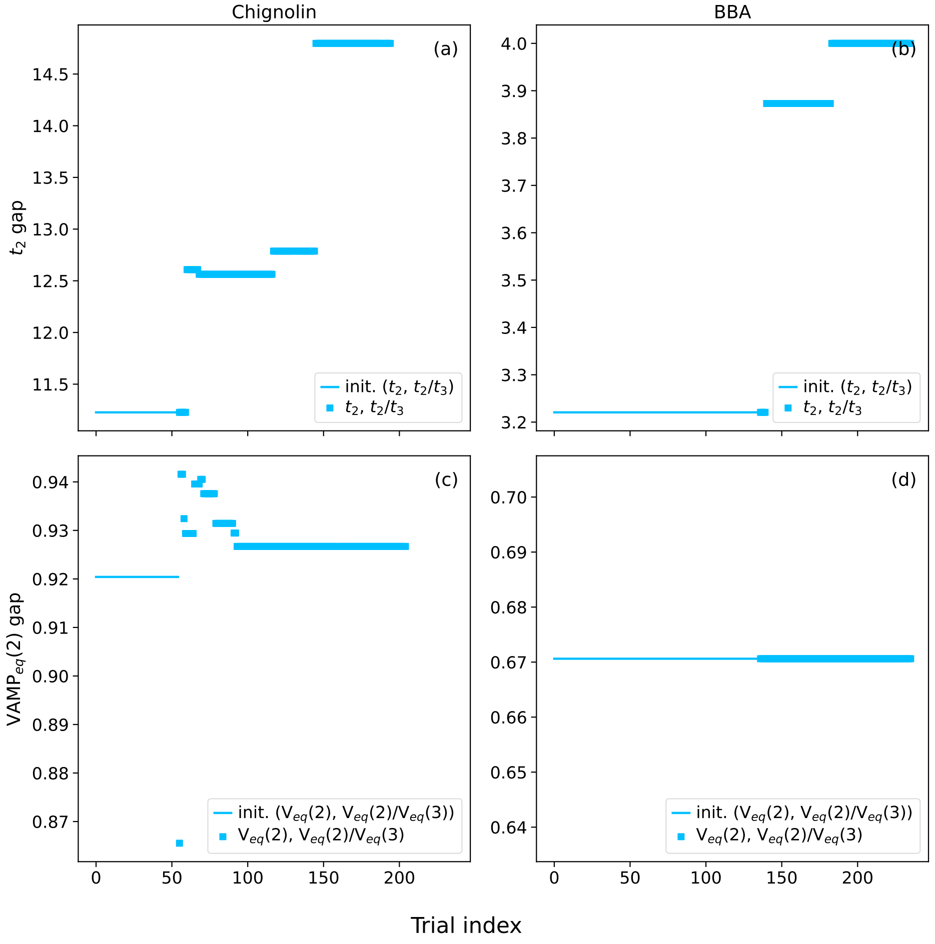

Figure 33: OPTIMISATION OF THE TIMESCALE AND VAMP2$_{eq}$ GAP. The blue dots show the incumbent gap during optimisation (shown only for the multi-objective optimisation). Panels (a) and (b) refer to the timescale gap t_2/t_3 while panels (c) and (d) refer to the VAMP2$_{eq}$(2)/VAMP2$_e q$(3). Panels (a) and (c) are for Chignolin optimisation while the panels (b) and (d) refer to BBA.

VAMP2$_{eq}$ vs Lag time.

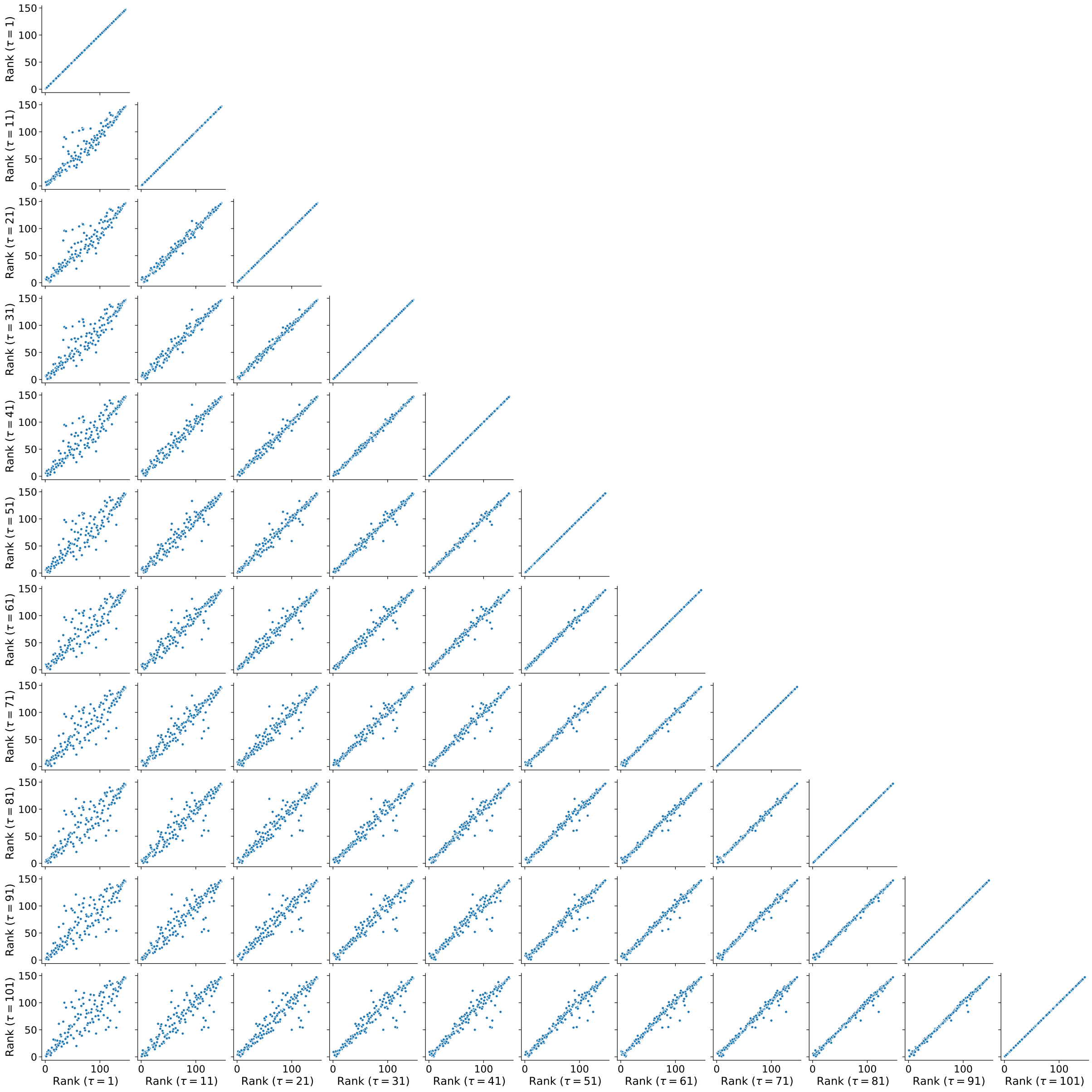

Figure 34: Pair plot of VAMP2$_{eq}$($k = 2$) rank with different lag times. The panel at position $(0, 1)$ plots the rank according toVAMP2$_{eq}$(2) against the rank according toVAMP2$_{eq}$(3), and similarly for other positions. This

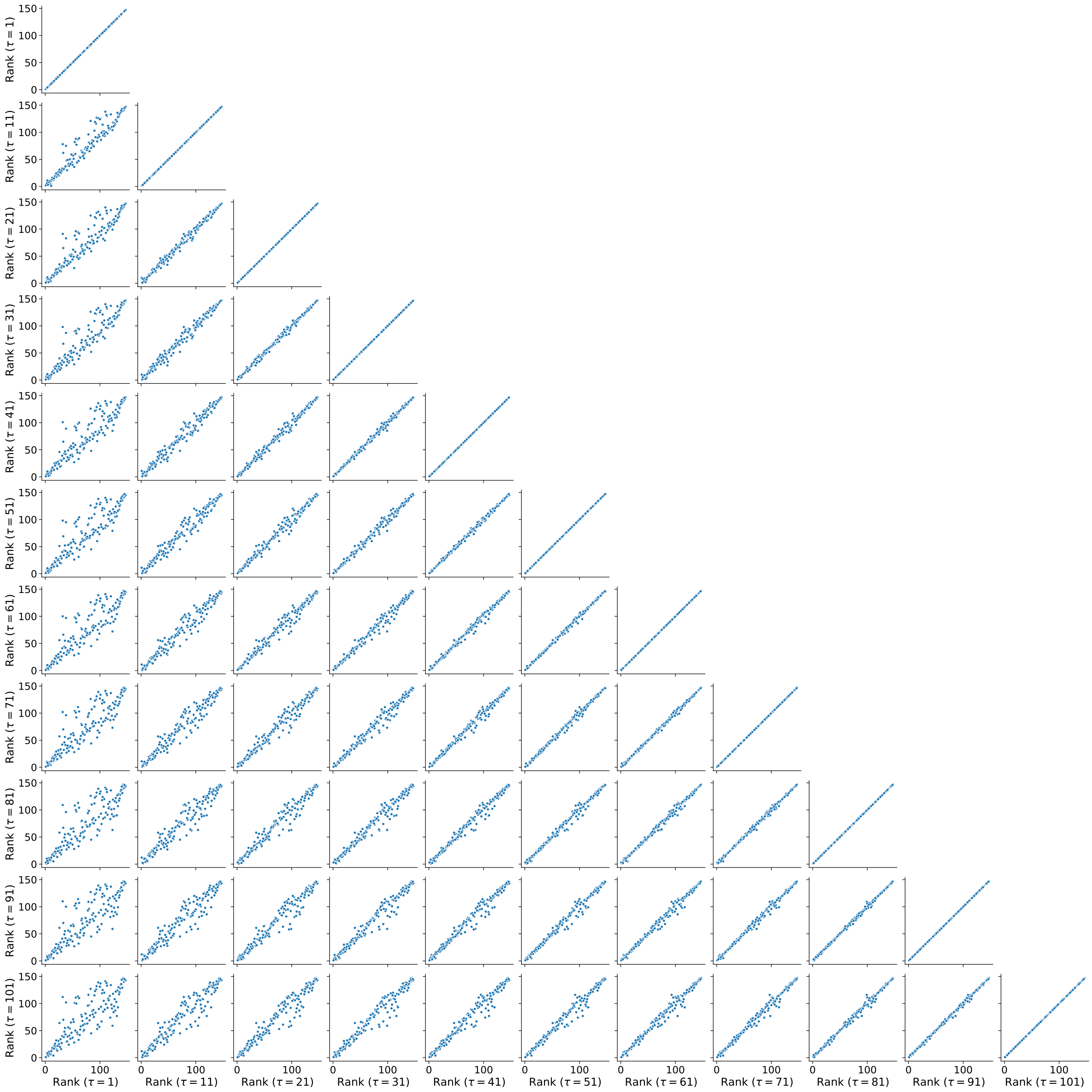

Figure 35: Pair plot of VAMP2$_{eq}(k = 3)$ rank with different lag times. The panel at position $(0, 1)$ plots the rank according toVAMP2$_{eq}(2)$ against the rank according toVAMP2$_{eq}(3)$, and similarly for other positions. This

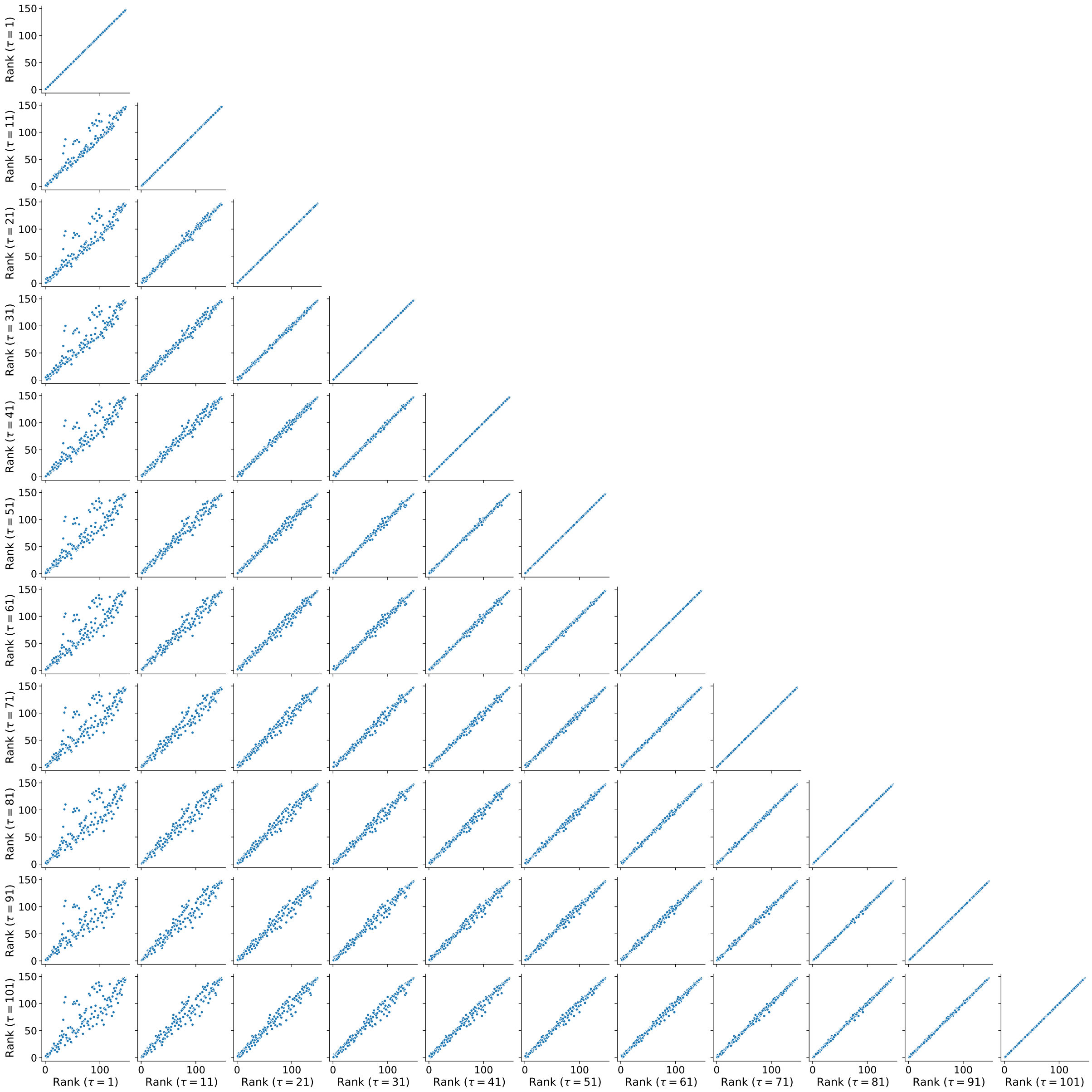

Figure 36: Pair plot of VAMP2$_{eq}$($k = 5$) rank with different lag times. The panel at position $(0, 1)$ plots the rank according toVAMP2$_{eq}(2)$ against the rank according toVAMP2$_{eq}(3)$, and similarly for other positions. This

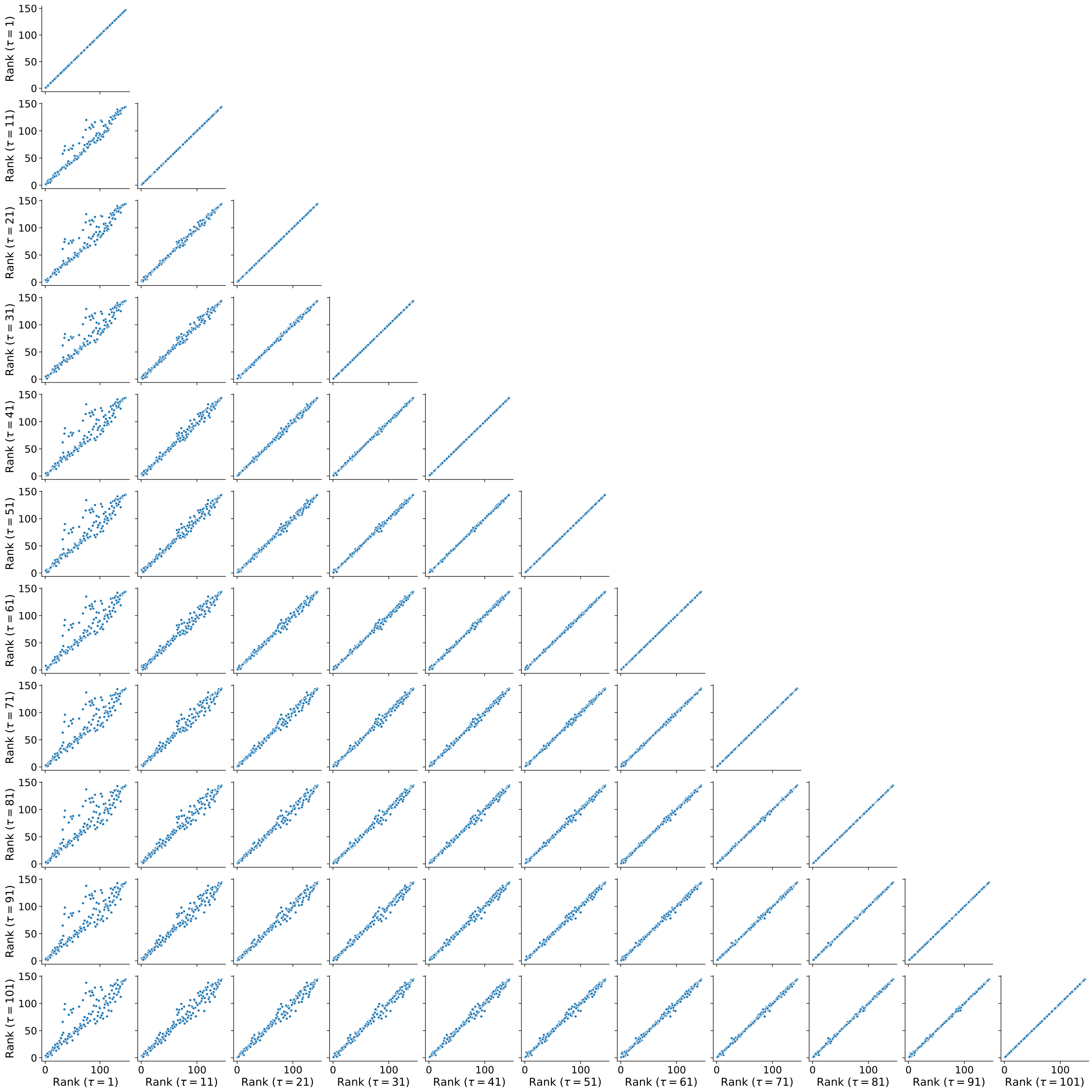

Figure 37: Pair plot of VAMP2$_{eq}(k = 10)$ rank with different lag times. The panel at position $(0, 1)$ plots the rank according toVAMP2$_{eq}(2)$ against the rank according toVAMP2$_{eq}(3)$, and similarly for other positions. This
